# Automated prediction of ground state spin for transition metal complexes†

**DOI:** 10.1039/d4dd00093e

**Published:** 2024-07-12

**Authors:** Yuri Cho, Ruben Laplaza, Sergi Vela, Clémence Corminboeuf

**Affiliations:** a Laboratory for Computational Molecular Design, Institute of Chemical Sciences and Engineering, École Polytechnique Fédérale de Lausanne Lausanne Switzerland clemence.corminboeuf@epfl.ch; b National Centre for Computational Design and Discovery of Novel Materials (MARVEL), École Polytechnique Fédérale de Lausanne Lausanne Switzerland; c National Centre for Competence in Research-Catalysis (NCCR-Catalysis), École Polytechnique Fédérale de Lausanne Lausanne Switzerland; d Departament de Ciència de Materials i Química Física and IQTCUB, Universitat de Barcelona Barcelona Spain; e Institut de Química Avançada de Catalunya (IQAC-CSIC) Barcelona Spain

## Abstract

Exploiting crystallographic data repositories for large-scale quantum chemical computations requires the rapid and accurate extraction of the molecular structure, charge and spin from the crystallographic information file. Here, we develop a general approach to assign the ground state spin of transition metal complexes, in complement to our previous efforts on determining metal oxidation states and bond order within the *cell2mol* software. Starting from a database of 31k transition metal complexes extracted from the Cambridge Structural Database with *cell2mol*, we construct the TM-GSspin dataset, which contains 2063 mononuclear first row transition metal complexes and their computed ground state spins. TM-GSspin is highly diverse in terms of metals, metal oxidation states, coordination geometries, and coordination sphere compositions. Based on TM-GSspin, we identify correlations between structural and electronic features of the complexes and their ground state spins to develop a rule-based spin state assignment model. Leveraging this knowledge, we construct interpretable descriptors and build a statistical model achieving 98% cross-validated accuracy in predicting the ground state spin across the board. Our approach provides a practical way to determine the ground state spin of transition metal complexes directly from crystal structures without additional computations, thus enabling the automated use of crystallographic data for large-scale computations involving transition metal complexes.

## Abstract 2

We develop a general approach to predict the ground state spin of transition metal complexes directly from crystal structures with 98% accuracy, thus enabling the automated use of crystallographic data in large-scale quantum chemical computations.

## Graphical abstract

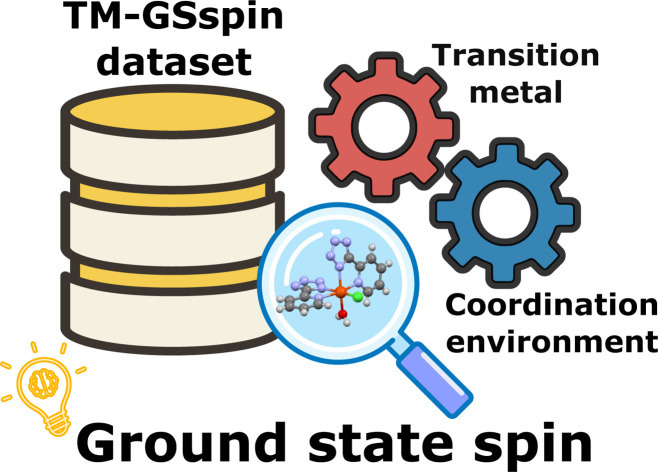

## 1 Introduction

The automated construction of datasets has become increasingly relevant for data-driven computational chemistry.^1–3^ Data-driven approaches to computational chemistry essentially include high-throughput screening of molecules and materials by quantum chemical (QC) computations^4–9^ as well as the use of large-scale computed data to train machine learning (ML) models for property prediction.^10–19^ Both tasks rely on extensive datasets curated to cover vast and diverse regions of chemical space.^20–22^ Within this context, crystallographic data repositories constitute a valuable pool of synthesized structures available in large size and chemical diversity.^23–26^ The Cambridge Structural Database (CSD)^27,28^ contains, for instance, over a million of experimental crystal structures collected over several decades.

Yet, the proper exploitation of crystallographic information for the computational chemistry is not straightforward. For datasets to be adapted for both the training of ML models and high-throughput QC searches, they must include the essential information needed to run an electronic structure computation, such as the structure (R), the molecular charge (Q) as well as the spin multiplicity. Owing to the lack of information about metal oxidation (OS)^29^ and spin states, reliably retrieving the molecular charge and spin multiplicity is especially difficult when transition metals (TM) are involved. To overcome this limitation, we recently developed *cell2mol*,^22^ a software that specifically interprets crystallographic data to retrieve the Cartesian coordinates, total charges, and connectivity of all individual molecules in the unit cell, including the OS of metal ions. While *cell2mol* provides a thorough unit cell interpretation, the original version was not coded to characterize ground state spins.

Given that the ground state spin of TM complexes depends on multiple factors, such as metal identity, OS, coordination geometry and ligand field strength, deducing this information only from their structure is challenging.^30–32^ Significant efforts have been made to train ML models that predict spin-state-dependent properties such as spin-splitting energies, spin-state orderings, sensitivity to Hartree–Fock exchange, and metal–ligand bond lengths^25,33–35^ but these efforts have been essentially placed on mononuclear octahedral complexes and on a restricted range of exemplary ligands with varying field strengths along the spectrochemical series. So far, the prediction of ground state spin of TM complexes has not been investigated across diverse chemical spaces.

**Fig. 1:**
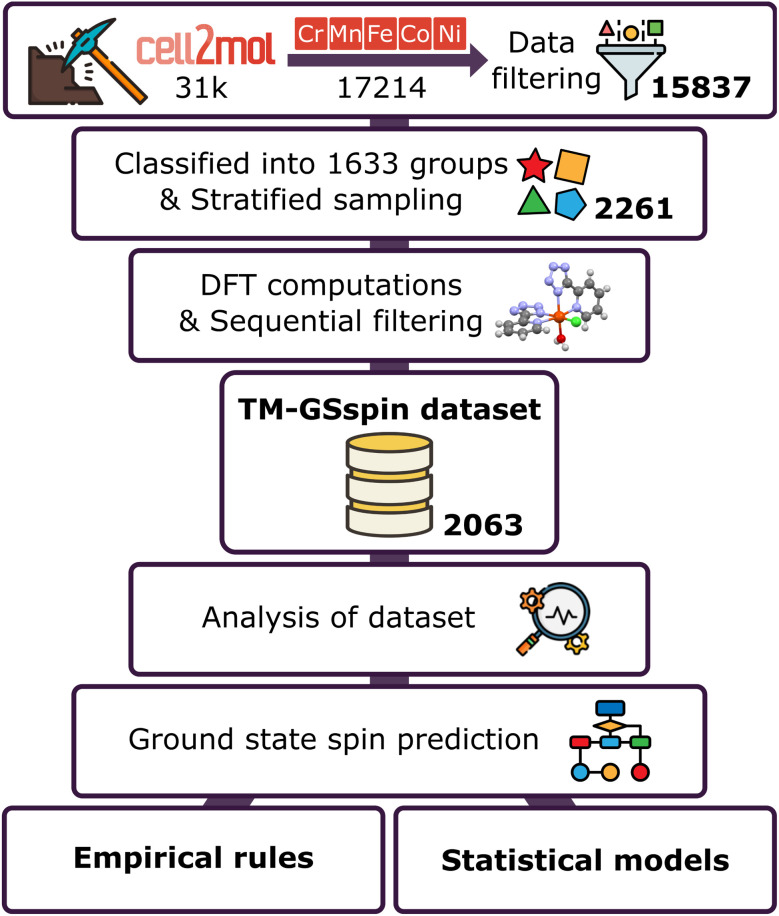
Proposed general workflow. The numbers in bold indicate the number of complexes curated at each step.

Herein, we develop a pragmatic and general workflow (Fig. 1) to predict the ground state spin of TM complexes by leveraging previously curated data obtained with *cell2mol*. Starting from the original database that was extracted from the CSD, we construct a smaller, representative, albeit diverse set through stratified sampling. We then determine the ground state spin of each individual complex through density functional theory (DFT) computation using the B3LYP* functional^36,37^ and filter out ambiguous cases. The resulting TM-GSspin dataset is systematically analyzed to identify correlations between the structural and electronic features of the complexes and their ground state spins. Based on the extracted patterns and relationships, we construct rule-based empirical and interpretative random forest models for ground state spin assignment in first row TM complexes. These models are integrated into *cell2mol*, enabling the assignment of total charge, OS, and ground state spin of TM complexes directly from crystallographic information files.

## 2 Methods

### 2.1 Dataset generation

Emphasis is placed on first row TM complexes with d electron configurations ranging from d^4^ to d^8^ that adopt different spin states depending on the nature of the metal and its coordination environment. Note that second and third row TM complexes are less cumbersome, as they typically exhibit low spin configurations due to larger crystal field splitting.^38,39^ As a starting point, we took the database containing 31k transition metal complexes we extracted from the CSD (updated May 2021) using *cell2mol* version 1.1.0.^22^ This database^22^ excludes polynuclear complexes, for which the total spin assignment would depend upon the coupling between spin-bearing metal centers, and complexes with formally radical ligands.

This results in 17 214 mononuclear first row complexes with five metal centers: Cr, Mn, Fe, Co, and Ni. Among these complexes, we excluded those with haptic ligands (*e.g.*, cyclopentadienyl) because their coordination numbers and geometries are ambiguous, presenting subtly different *η* coordination modes.^40^ We also eliminated complexes with nitrosyl ligands to avoid potential spin from typical non-innocent ligands.^41^ The coordination geometry of the TM complex was then determined using the CoSymLib python library^42^ and complexes exhibiting significant deviation from the ideal shape of a reference polyhedron were removed to unequivocally identify the correlation between the ground state spin and the coordination geometry (see Section S1 and Fig. S1 in the ESI†).

The remaining 15 837 complexes were classified based on metal identity, OS, coordination number (the number of atoms bound to the metal center), coordination geometry, and composition of the metal-coordinating atoms, resulting in 1633 distinct groups of complexes that share the aforementioned characteristics. We then performed stratified sampling among those groups to construct a dataset of 2261 complexes where each group is represented (see Section S1 in the ESI† for further details regarding dataset construction and curation, and Section S2† for a discussion on the excluded complexes).

### 2.2 Ground state spin computations

Crystal structure geometries were refined by optimizing the atomic positions of hydrogen atoms in either the singlet or doublet state, as hydrogen atoms generally exhibit the greatest uncertainty in refinement due to their small electron density. Optimizations were carried out using Gaussian09 (revision D.01)^43^ at the B3LYP*-D3(BJ)/def2-SVP level.^44,45^ B3LYP* is a reparametrized version of B3LYP that reduces Hartree–Fock exchange from 20% to 15%, and was shown to improve the description of spin-splitting energetics in TM complexes.^36,37^ Single point computations were then performed at the B3LYP*-D3(BJ)/def2-TZVP level for three different spin states: singlet, triplet, and quintet for systems with an even number of electrons, and doublet, quartet, and sextet otherwise. The spin state with the lowest energy was assigned as the ground state spin. B3LYP* results were compared to two other functionals, TPSSh and M06L, which have also demonstrated good performance in describing splitting energies in spin-crossover Fe complexes.^46,47^ Except for sensitive cases (*vide infra*), consistent ground state spins were observed with all three levels (see Fig. S3 in the ESI†).

An iterative and automated correction was applied in case of convergence failures. Complexes for which computations failed to converge in all accessible spin states were removed. Those with energy gaps between possible spin states smaller than 5 kcal mol^−1^ were excluded, as they fall below the chemical accuracy of DFT for spin-state energetics of TM complexes.^48–50^ This filtering step also eliminates spin-crossover complexes, for which ground spin state changes with an external stimulus like temperature or pressure as a result of vibrational and electronic entropy contributions.^33,46,51,52^ Finally, complexes exhibiting an expectation value of 〈*Ŝ*^2^〉 that deviates from the exact value of *S*(*S* + 1) by more than 0.1 for the singlet and doublet ground states, and more than 0.2 for the other ground state spins^49^ were also excluded. Overall, a total of 198 complexes were removed by the three consecutive filters. For further details on the in-depth analysis of the DFT results, excluded complexes, impact of geometry optimization on spin-splitting energies, and complexes with hydride ligands, see Section S2 in the ESI.†

## 3 Results and discussion

### 3.1 TM-GSspin dataset

The final curated TM-GSspin dataset consists of 2063 mononuclear complexes and their corresponding ground state spins. The following subsections analyze the dataset and more specifically the relationship between structural and electronic features of the complexes and their ground state spins.

#### 3.1.1 Chemical diversity of the dataset

**Fig. 2:**
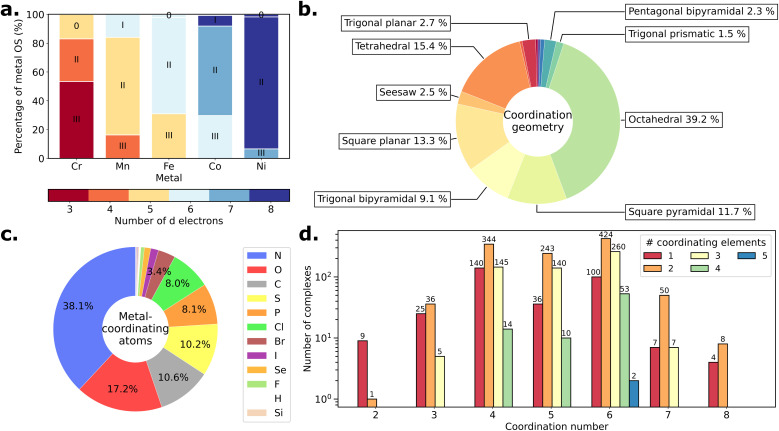
Chemical diversity of the TM-GSspin dataset. (a) Percentage of oxidation state (OS) for each metal. The OS is denoted 0, I, II, or III. The color code indicates the number of d electrons in the metal ion. (b) Frequency distribution of coordination geometries. (c) Frequency distribution of metal-coordinating atom elemental identities. Elements with a frequency < 0.2% are omitted in the legend box. (d) Number of different elements in the first coordination sphere for each coordination number.

Fig. 2 illustrates the large chemical diversity of the TM-GSspin dataset, encompassing various metal identities, OS, coordination geometries, as well as compositions of the first coordination sphere. Each metal exhibits three OSs, with a number of d electrons ranging from 3 to 8 (Fig. 2a). Eighteen types of coordination geometries are obtained, with coordination numbers ranging from 2 to 8 (Fig. 2b). Those include common geometries like octahedral, tetrahedral, or square planar, as well as less common ones such as linear, trigonal planar, pentagonal bipyramidal, or capped trigonal prismatic. Nitrogen is found to be the most recurrent metal-coordinating atoms followed by oxygen, carbon, sulfur, phosphorus, chlorine, and bromine (Fig. 2c). Combinations of different metal-coordinating elements across various coordination numbers and geometries lead to over 600 different first coordination spheres, including complexes with up to 5 different coordinating elements (Fig. 2d). In comparison with the original database^22^ of 15 837 complexes mined from the CSD, the TM-GSspin dataset is characterized by an increased proportion of complexes with less common coordination geometries (*e.g.*, the proportion of trigonal planar complexes increases from 0.7% in the original database to 2.7% in the TM-GSspin dataset) and those with zero or low-valent OSs (see comparison in Fig. S2 in the ESI†). Additionally, Fig. S6 and S7 in the ESI† provide an overview of the TM-GSspin dataset, including the number of atoms, number of electrons, total molecular charges, and spin multiplicity, as well as the sizes of the ligands.

#### 3.1.2 Analysis of the transition metal complexes ground state spins

**Fig. 3:**
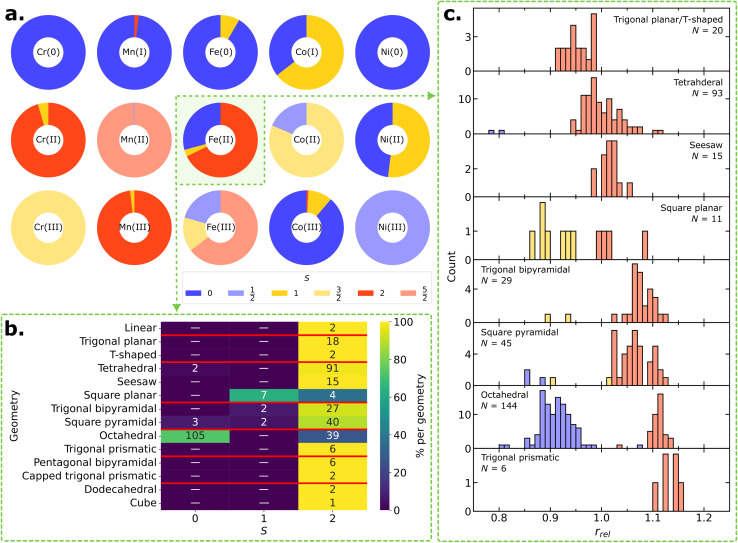
Relationship between ground state spins and various features of TM complexes. (a) Proportion of ground state spins for different metal centers and oxidation states. (b) Ground state spins of d^6^ Fe(ii) complexes based on their coordination geometries. Each column represents a total spin quantum number *S*, and each row represents a coordination geometry. Red horizontal lines classify coordination geometries by their coordination numbers. The number within each grid cell indicates the number of corresponding complexes in that cell (0 is shown as a hyphen). The color code represents the proportion of spin states within a given coordination geometry, where navy blue corresponds to 0% and yellow to 100%. (c) Histograms of relative metal radii (*r*_rel_) and corresponding ground state spins for Fe(ii) complexes with coordination numbers ranging from 3 to 6. *N* indicates the number of complexes used to plot each histogram. The color code represents the ground state spin: blue (singlet), yellow (triplet), and red (quintet).

Fig. 3a shows the proportion of ground state spin of TM complexes for each metal and OS. Some metals in a given OS consistently exhibit the same ground state spin. As expected, if the number of d electrons is less than 4 or more than 8, the number of unpaired d electrons is the determining factor. For instance, all Cr(iii) complexes (d^3^ configuration) have a quartet ground state. Alternatively, zero–valent complexes such as Cr(0), Fe(0), or Ni(0) centers as well as and Mn(i) complexes possess a singlet ground state. These complexes typically bind to strong-field ligands such as carbonyls or substituted phosphines, which cause a substantial energy separation between d orbitals and favor lower spin states. Intriguingly, one Fe(0) tetrahedral complex (CSD refcode: NUNWUP^53^) and one Mn(i) linear complex (CSD refcode: CUJSAD^54^) deviate from this trend (Fig. S8 in the ESI†). The behavior of the former can be attributed to eight valence electrons in the tetrahedral crystal field, while the linearity in the latter, reported as [K(15-crown-5)_2_][Mn{C(SiMe_3_)_2_}], is affected by crystal packing effects.^54^ The d^7^ Ni(iii) complexes, in the dataset, constitute another constant example that exclusively adopt the doublet ground state.

TM centers exhibiting various ground state spins can be understood based on their coordination environments. As an illustrative example, the analysis of the ground state spins of Fe(ii) complexes across fourteen different coordination geometries is provided in Fig. 3b d^6^ Fe(ii) complexes adopt singlet, triplet, or quintet ground state, which corresponds to low-spin (LS), intermediate-spin (IS), or high-spin (HS) state, respectively. Most coordination geometries of these complexes exhibit the HS ground state. Specifically, all Fe(ii) complexes with coordination numbers smaller than 4 or greater than 6 (*i.e.*, linear, trigonal planar, T-shaped, pentagonal bipyramidal, capped trigonal prismatic, dodecahedral and cube geometries) are consistently HS. Tetrahedral, seesaw, and trigonal prismatic Fe(ii) complexes are all HS except for two tetrahedral imido complexes containing tertiary phosphine ligands, which adopt LS states. Fe(ii) complexes with other coordination geometries exhibit a greater ground state spin variability. Square planar and trigonal bipyramidal Fe(ii) complexes exhibit IS or HS, depending on whether the highest d orbital is empty or half-filled. Square pyramidal Fe(ii) complexes cover three different ground state spins, while most common octahedral Fe(ii) complexes adopt either the LS or HS ground states.

Similar trends are observed for other TM complexes with d^4^ to d^8^ electron configurations (see in Fig. S9–S13 of the ESI†). Complexes with low- or high-coordination geometries tend to favor the HS state within a given d electron configuration. Certain coordination geometries, such as tetrahedral and trigonal prismatic, exclusively adopt the HS states due to the small d-orbital splitting in these crystal fields. However, a few tetrahedral complexes favor the LS ground state owing to the presence of strong-field ligands such as substituted phosphines. Complexes with other coordination geometries exhibit different ground state spins depending on the arrangement of d electrons within a given crystal field, which indicates that considering additional factors is crucial for determining the ground state spin. For further analysis, the relationship between ground state spins and coordination sphere compositions of 144 Fe(ii) octahedral complexes are shown in Fig. S14 in the ESI,† with a brief discussion.

We further examine the distribution of distances between the metal center and its coordinating atoms as the metal–ligand bond lengths in HS states are generally longer compared to those in the LS states due to the population of anti-bonding orbitals. Within this context, Taylor *et al.*,^25^ assigned ground state spins of mononuclear octahedral Fe(ii)/Fe(iii) complexes based on heuristic cut-off values for metal–ligand bond lengths. We here introduce a more general indicator applicable to various metal centers and coordination geometries. We define the relative metal radius, denoted as *r*_rel_, as1
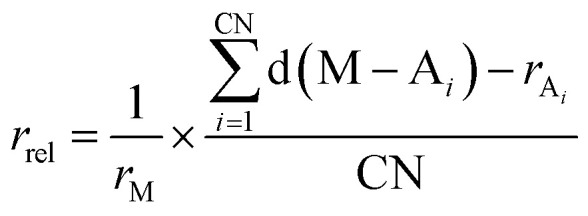
where CN is the coordination number of a given complex, d(M − A_*i*_) represents the distance between a metal center (M) and a coordinating atom (A_*i*_), and *r*_M_ and *r*_A_*i*__ are covalent radii of M and A_*i*_, respectively. Covalent radii values were taken from a previous analysis of experimental crystal structures (Table S8 in the ESI†).^55^

Fig. 3c shows the distribution of relative metal radii and their corresponding ground state spins for Fe(ii) complexes depending on the coordination geometry (see Fig. S15 in the ESI† for Fe(iii) complexes). Across different coordination geometries, Fe(ii) complexes in the HS state typically possess longer relative metal radii compared to those in the LS or IS states. In general, we observe a biased distribution toward HS states with longer relative metal radii. In particular, the relative metal radii of octahedral Fe(ii) complexes show a binomial distribution, separating LS and HS. Interestingly, two tetrahedral Fe(ii) complexes with LS state exhibit very short relative metal radii, which deviate from the overall distribution of relative metal radii in tetrahedral complexes. This suggests that relative metal radius serves to identify outliers exhibiting uncommon ground state spins. For further analysis, we investigate one octahedral singlet complex (CSD refcode: DOQRAC) with longer relative metal radii in Fig. 3c, which was identified as a spin-crossover complex in the literature.^56^ Moreover, there is a systematic increase in relative metal radius as the coordination number increases. This pattern is especially evident in HS complexes, which display a linear increase, as shown in Fig. S16 in the ESI.†

### 3.2 Ground state spin assignment based on empirical rules

**Fig. 4:**
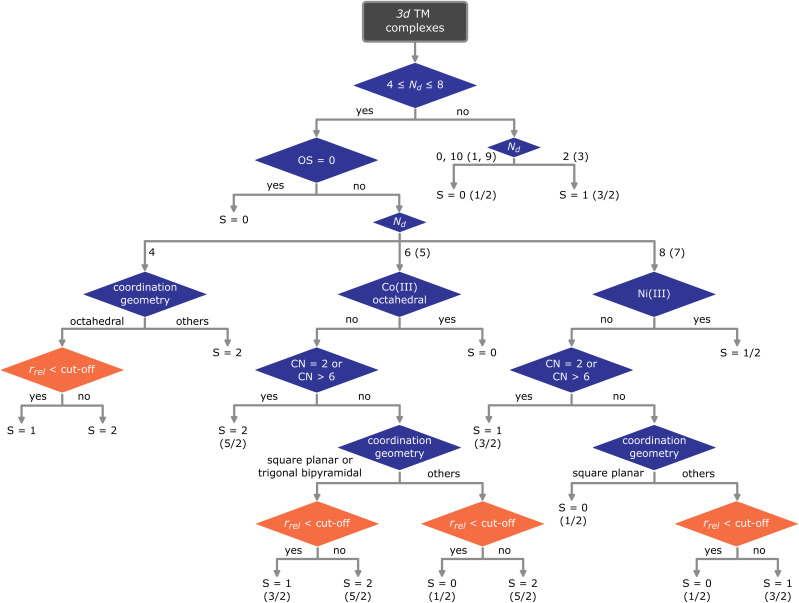
Ground state spin assignment of first row TM complexes based on empirical rules. *N*_d_: the number of d electrons, with odd numbers of d electrons shown in parentheses, OS: metal oxidation state, CN: coordination number, *r*_rel_: relative metal radius, cut-off: predefined cut-off value used to distinguish the lowest spin state. Decision nodes in orange use cut-off values that are specific for a given metal and coordination geometry (see Table S10 in the ESI†).

Based on the trends and relationships observed in the TM-GSspin dataset, we develop an empirical model to assign the most probable ground state spin for first row TM complexes. Fig. 4 shows a rule-based decision tree used in our assignment model, which systematically considers key structural and electronic features of the complexes. The decision tree begins by considering two key factors: the number of d electrons and the OS of the metal center. For coordination complexes with 0, 1, 2, 3, 9, or 10 d electrons, the ground state spin is determined based on the number of unpaired electrons. For complexes with d electrons between 4 and 8 (excluding zero-valent complexes), the decision tree takes into account the coordination environments. Note that d^6^ Co(iii) octahedral (Fig. S11†) and d^7^ Ni(iii) complexes (Fig. S12†) exclusively exhibit the LS state, regardless of their coordination environments. Therefore, their ground state spins are assigned as singlet and doublet, respectively.

For complexes with a coordination number of 2 or greater than 6, the ground state spin is assigned as HS based on observed trends. We hypothesize that in such low- or high-coordination cases, the ligand field is weak due to the limited interaction between the metal and ligands. This stems from either sterically hindered bulky ligands in the former case (Table S9 in the ESI†) or overly crowded coordination spheres in the latter. Accordingly, both extremes favor the HS ground state.

For the remaining complexes, the assignment depends on their coordination geometry and relative metal radius. For cases with multiple ground state spins within a given coordination geometry, the model uses the relative metal radius as a distinguishing criterion (indicated by the orange rhombus in Fig. 4). For this step, we define a specific cut-off for each combination of metal and coordination geometry (Table S10 in the ESI†). If the relative metal radius falls below the designated cut-off value, the ground state spin is assigned as the lowest possible spin state. Note that for d^4^ octahedral as well as d^6^ square planar or trigonal bipyramidal complexes, the lowest spin state is assigned as triplet based on the pattern discerned in the dataset.

Despite its relative simplicity, the empirical model achieves a high 97% accuracy within the dataset. Out of the 2063 complexes considered, only 55 exhibit discrepancies in their ground state spin assignments with respect to the computations. Most of the disagreements occur for square pyramidal complexes featuring Fe(iii), Co(ii), and Ni(ii) with relative metal radii close to the cut-off values. Furthermore, our empirical model is indeed unable to assign the IS state to square pyramidal Fe complexes, which would require an additional cut-off value.

### 3.3 Ground state spin prediction with statistical models

**Fig. 5:**
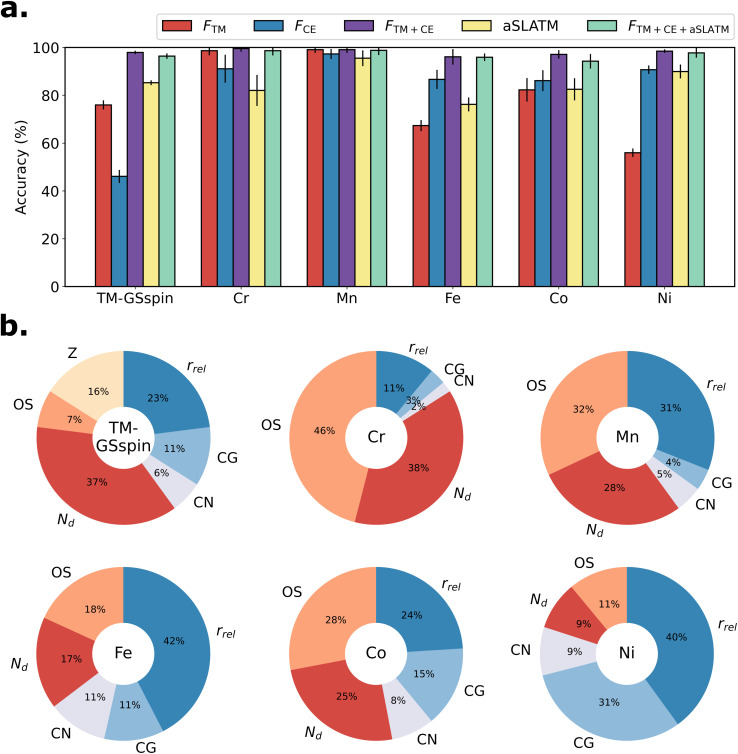
Ground state spin prediction for the TM-GSspin dataset and for each metal subset. (a) 10-Fold cross-validated accuracy of random forest models using different features. *F*_TM_ is a vector containing the metal atomic number (*Z*), metal OS, and the number of d electrons (*N*_d_). *F*_CE_ contains coordination number (CN), coordination geometry (CG), and relative metal radius (*r*_rel_). *F*_TM+CE_ combines both *F*_TM_ and *F*_CE_. aSLATM is the atomic SLATM representation^57^ of the metal atom. *F*_TM+CE+aSLATM_ incorporates *F*_TM+CE_ and aSLATM. (b) Feature importances in prediction models trained using *F*_TM+CE_.

As an alternative to using simple empirical rules, we train statistical models for ground state spin prediction, using combinations of features used in the empirical models as part of the input vectors. First, we construct a feature vector *F*_TM_, containing only information about the metal center: the metal atomic number, the metal OS, and the number of d electrons. The second vector *F*_CE_ contains only geometric features of the coordination environment: coordination number, coordination geometry, and relative metal radius. Third, *F*_TM+CE_ incorporates both *F*_TM_ and *F*_CE_. For comparison, we include the atomic Spectrum of London and Axilrod–Teller–Muto potential (aSLATM) physics-based representation^57^ by using a vector that concatenates one, two, and three-body terms associated with the TM as implemented in the QML package^58^ with a modified grid and cutoff consistent with our previous work.^22^ Finally, *F*_TM+CE+aSLATM_ concatenates *F*_TM+CE_ and aSLATM. We employ random forest models trained on the TM-GSspin dataset using all feature vectors. We also train models on individual metal subsets to assess the impact of each metal separately. An overview of the performance of these models is presented in Fig. 5.

Fig. 5a shows the 10-fold cross-validated accuracy for each random forest model, with error bars representing the standard deviation across folds. Amongst the models trained on the entire TM-GSspin dataset (Fig. 5a, leftmost), the model using *F*_TM+CE_ achieves the highest cross-validated accuracy, reaching 98%. In contrast, the model with the *F*_CE_ features exhibits the poorest performance due to the lack of metal center information. *F*_CE_ fails to capture the intricate relationship between ground state spin and coordination geometry, which varies depending on the TM and its OS. For comparison, the model employing aSLATM outperforms the one based on *F*_CE_, benefiting from the inclusion of nuclear charge information for the metal atom. Interestingly, the *F*_TM+CE_ features lead to a more accurate model than aSLATM despite its simplicity. This superiority is attributed to *F*_TM+CE_ explicitly containing electronic information, such as the number of d electrons and metal OS—critical factors closely linked to the ground state spin of the complex, which are not explicitly captured by many-body potential terms. When considering standard deviations, the model using *F*_TM+CE_ displays an accuracy similar to the model using the much larger *F*_TM+CE+aSLATM_ (vector size 6 *vs.* 80 591) while bypassing the computational cost of generating the aSLATM representation. Overall, these results underscore the effectiveness of *F*_TM+CE_ in capturing both the electronic and structural information of TM complexes, crucial for determining their ground state spin. Separately, we performed dimensionality reduction on the aSLATM by using principal component analysis to reduce the number of features to 100. The reduced aSLATM resulted in slightly worse performance compared to the original aSLATM, as shown in Table S12 in the ESI.†

The performance of the models trained on the individual TM subset are also shown in Fig. 5a. The models using the *F*_TM_ features exhibit high accuracy for Cr and Mn complexes, owing to the strong relationship between the d electron configuration and the ground state spin for these elements. Conversely, for Fe, Co, and Ni complexes, using only *F*_CE_ outperforms the *F*_TM_ models, which is especially evident for the Ni complexes. The reason for the latter is that the majority of Ni complexes in the dataset are either Ni(ii) square planar or octahedral complexes, consistently displaying a singlet or triplet ground state, respectively. For these individual metal subsets, models using *F*_CE_ are comparable or even more accurate than those using aSLATM, contrasting with the trends obtained for the corresponding models trained on the entire TM-GSspin dataset. This distinction arises because the relationship between the ground state spin and the coordination environment is well-defined within each metal subset but not on the overall dataset, in which each metal exhibits different preferences. Ultimately, the best performance obtained on the individual subsets is consistently achieved for the models employing *F*_TM+CE_.

To shed light on the relevance of the various features, we finally examine the feature importance derived from the *F*_TM+CE_ random forest models, as shown in Fig. 5b. Overall, the relative metal radius (*r*_rel_) and the number of d electrons (*N*_d_) emerge as the most influential factors. In agreement with our previous observations (*vide supra*), predictions for Cr and Mn complexes primarily rely on features associated with the metal center, while predictions for Ni complexes are driven by geometric information. Nevertheless, even in those cases, the incorporation of both electronic and structural descriptors remains crucial to predict the ground state spins of TM complexes in both individual metal subsets or full dataset. For more comprehensive information regarding the performance of random forest models, a detailed list of complexes with incorrect predictions, and the analysis of feature importances in models trained using *F*_TM+CE+aSLATM_, see Tables S13, S14, and Fig. S18 in the ESI.†

## 4 Conclusion

In order to facilitate the high-throughput generation of computed data by retrieving information from existing crystallographic data repositories, we here present ground state spin prediction models that will extend the capability of our *cell2mol*^22^ software. *cell2mol* was built to offer a comprehensive interpretation of the unit cell by extracting the connectivity and total charge of molecules within a crystal structure, placing emphasis on transition metal-containing structures. Yet, information regarding another necessary input of quantum chemical computations, *i.e.*, the molecular spin, was missing from the interpretation. Here, we propose a general approach to predict the ground state spin of TM complexes. The TM-GSspin dataset, comprising 2063 mononuclear first row TM complexes and their computed ground state spins, was constructed starting from the 31k complexes extracted from the CSD with *cell2mol*. TM-GSspin is currently the largest dataset that encompasses a diverse range of metals, metal OS, coordination geometries, and first coordination sphere compositions, while also containing total charge and ground state spin information.

The analysis of TM-GSspin uncovered correlations between ground state spins and the various features of TM complexes (*e.g.*, metal OS, the number of d electrons, coordination number, geometry, and the relative metal radius measure introduced herein). While most of the relationships were already established, we quantified their validity across the board and exploited them to build rule-based decision trees to assign the ground state spin for first row TM complexes. The most relevant features were also used as inputs of random forest models, achieving an impressive 98% cross-validated accuracy within the dataset.

These models are fully integrated into the latest version of *cell2mol* which is now capable of determining the total charge, the OS, and the ground state spin of TM complexes directly from crystallographic data. This work streamlines automation of electronic structure workflows of molecules extracted from crystal structure repositories.

## Data availability

The TM-GSspin dataset as well as the additional complexes discussed in the ESI† are available in the Materials Cloud Repository https://doi.org/10.24435/materialscloud:jx-a5. The ground state spin prediction models are available in the development version of *cell2mol*https://github.com/lcmd-epfl/cell2mol/tree/dev. Random forest models are trained and tested by using the Python script https://github.com/lcmd-epfl/cell2mol/blob/dev/cell2mol/random_forest.py.

## Author contributions

Y. C. and C. C. conceived the project. Y. C. curated the dataset, performed the DFT computations, constructed empirical and statistical models, and analyzed the results with help from R. L. and S. V. All authors discussed the results. The original manuscript was written by Y. C. with help and feedback from all authors. C. C. provided supervision throughout and was responsible for funding acquisition.

## Conflicts of interest

There are no conflicts to declare.

## Supplementary Material

DD-003-D4DD00093E-s001
