# Supplementary material for: Automated prediction of ground state spin for transition metal complexes
Source: Digit Discov. 2024 Jul 12;3(8):1638–47. doi: 10.1039/d4dd00093e (PMC11305380; doi:10.1039/d4dd00093e)
Supplement: DD-003-D4DD00093E-s001 [file DD-003-D4DD00093E-s001.pdf]

Electronic Supplementary Material (ESI) for Digital Discovery.
This journal is © The Royal Society of Chemistry 2024

Supporting Information: Automated Prediction of Ground State Spin for Transition Metal Complexes

Yuri Cho,[†,‡] Ruben Laplaza,[†,¶] Sergi Vela,[§,‖] and Clémence Corminboeuf*,[†,‡,¶]

†*Laboratory for Computational Molecular Design, Institute of Chemical Sciences and Engineering, École Polytechnique Fédérale de Lausanne (EPFL), Lausanne, Switzerland*

‡*National Centre for Computational Design and Discovery of Novel Materials (MARVEL), École Polytechnique Fédérale de Lausanne (EPFL), Lausanne, Switzerland*

¶*National Centre for Competence in Research–Catalysis (NCCR–Catalysis), École Polytechnique Fédérale de Lausanne (EPFL), Lausanne, Switzerland*

§*Departament de Ciència de Materials i Química Física and IQTCUB, Universitat de Barcelona, Barcelona, Spain*

‖*Institut de Química Avançada de Catalunya (IQAC-CSIC), Barcelona, Spain*

E-mail: clemence.corminboeuf@epfl.ch

Contents

List of Figures **S-2**

List of Tables **S-3**

S1 Dataset generation **S-5**

S2 Ground state spin computations **S-10**

S3 TM-GSspin dataset **S-21**

S4 Ground state spin assignment based on empirical rules **S-27**

S5 Ground state spin prediction with statistical models **S-31**

References **S-35**

List of Figures

S1 Distributions of deviation values from the determined coordination geometry S-7

S2 Chemical diversity of the original database of 15,837 complexes S-8

S3 Benchmark of DFT functionals B3LYP*, TPSSh, and M06L S-16

S4 Ground state spin of 3d, 4d, or 5d TM complexes with/without haptic ligands S-17

S5 Impact of geometry optimization on spin-splitting energy S-19

S6 An overview of TM-GSspin dataset S-21

S7 Sizes of the ligands in TM-GSspin S-21

S8 Ground state spin of zero-valent, Mn(I) and Ni(III) complexes S-22

S9 Ground state spin of d^4 Cr(II) and Mn(III) complexes. S-23

S10 Ground state spin of d^5 Mn(II) and Fe(III) complexes. S-23

S11 Ground state spin of d^6 Fe(II) and Co(III) complexes. S-23

S12 Ground state spin of d^7 Co(II) and Ni(III) complexes. S-24
S13 Ground state spin of d^8 Co(I) and Ni(II) complexes. S-24
S14 Ground state spins of Fe(II) octahedral complexes depending on coordination sphere composition S-25
S15 Histograms of relative metal radii and ground state spins for Fe(III) complexes S-26
S16 Relative metal radius depending on the coordination number S-28
S17 Relationship between relative metal radius and average of metal radius . . . S-28
S18 Feature importances in prediction models trained using $F_{\mathrm{TM+CE+aSLATM}}$ . . . S-31

List of Tables

S1 Subgroups in a group of octahedral Cr(0) complexes coordinating six carbon atoms based on the formula of ligands S-9
S2 Accessible spin states based on d^n electron configuration S-10
S3 Excluded complexes due to small energy gap between low-lying spin states . S-11
S4 Excluded complexes due to significant spin contamination in the ground state S-12
S5 Unrepresented groups in the TM-GSspin dataset S-13
S6 Ground state spin of nitrosyl complexes S-18
S7 Exemplary complexes to assess the impact of geometry optimization on spin-splitting energy S-19
S8 Covalent radii used in this work S-27
S9 2-coordinate complexes in TM-GSspin and their ligand information S-29
S10 Cut-off values for empirical models S-30
S11 Comparison of performances of random forest models using F_{CE} and F_{CE} plus the average number of atoms in ligands S-32
S12 Comparison of performances of random forest models using aSLATM and reduced aSLATM using PCA S-32

S13 Performance of random forest models for ground state spin prediction S-33
S14 Investigation of incorrect prediction S-34

S1 Dataset generation

The database[1] we previously extracted from the Cambridge Structural Database (CSD) contains 31,019 mononuclear complexes featuring eight different metal centers (Cr, Mn, Fe, Co, Ni, Cu, Ru, and Re), whose unit cells were successfully interpreted by *cell2mol*, and for which metal oxidation state (OS) matched the respective *.cif* file. Before creating this database, we performed pre-filtering to exclude complexes missing hydrogens. Moreover, this database does not include any formally radical ligand or polynuclear complexes. In this work, we focused on first row transition metal complexes with five metal centers Cr, Mn, Fe, Co, and Ni whose d electron configurations ranging from d^4 to d^8, which reduces the initial database from 31,019 to 17,214 entries.

Among those, 1,211 complexes identified by *cell2mol* as containing haptic ligands were excluded. We then checked the coordination number of the complexes, defined as the number of atoms bound to the metal center. For 650 complexes, the coordination numbers determined by *cell2mol* differ from those displayed in the CSD software Conquest. These discrepancies did not impact the assignment of metal OS. Still, a correction procedure, guided by the coordination environments and metal-ligand bond lengths, was applied to identify the metal-coordinating atoms. 17 complexes that showed a disagreement in the coordination number after correction were removed. 34 additional nitrosyl complexes were removed to avoid potential spin contributions from typical non-innocent ligands.[2]

The complexes' coordination geometry was assigned using a continuous shape measure, abbreviated as CShM, as implemented in the CoSymLib python library.[3] The CShM value is defined as the deviation from an ideal shape of a polyhedron, measured on a scale from 0 to 100. For each complex, the CShM values were obtained according to the selected reference shapes in the corresponding coordination number. We assign the coordination geometry to the shape characterized by the lowest CShM value. 115 complexes exhibiting very significant distortions and featuring a deviation value higher than 7.0 were excluded after this step. This selected threshold is chosen to be considerably higher than the rule of thumb values defined in

the literature,[4] in which CShM values below 1.0 are considered to indicate minor distortion from the reference shape, while values of up to 3.0 are considered to indicate important distortion. However, it is important to note that even for CShM values around or above 3.0, the reference shape still provides a reasonably good stereochemical description.[4] Figure S1 depicts the distributions of deviation values of the coordination geometry determined for complexes with coordination numbers ranging from 2 to 8. The deviation values, and the metal-coordinating atoms are given for several examples (*i.e.*, CSD refcodes LUMZEZ, OBIVAX, NODHUK, RAGLOD, REJPOM, VIDMIH, WUBFUX, BEJVAO, and ZIRKEQ).

The remaining 15,837 complexes constitute the "original database", which shows large chemical diversity as illustrated in Figure S2 but also large redundancy. We performed stratified sampling to construct a more compact, albeit representative dataset and avoid the cost of the ground state spin computations for redundant complexes. We labeled the original database of 15,837 complexes based on the following classifiers: metal identity, metal OS, coordination number, coordination geometry, and composition of metal-coordinating atoms. This resulted in 1,633 distinct groups, where the complexes within each group shared the same aforementioned characteristics. The group size ranges from 1 to 616. Within each group, we chose the complexes with the lowest total number of electrons to minimize computational cost. In cases where multiple complexes possess an identical minimal electron count, the one closest to the ideal reference shape was selected. To further enrich the dataset, 628 complexes were additionally selected from the groups consisting of more than one member, prioritizing the complexes with lower electron counts until at least 1 % of the group was selected. As a result, a total of 2,261 complexes were selected for the density functional theory (DFT) computations.

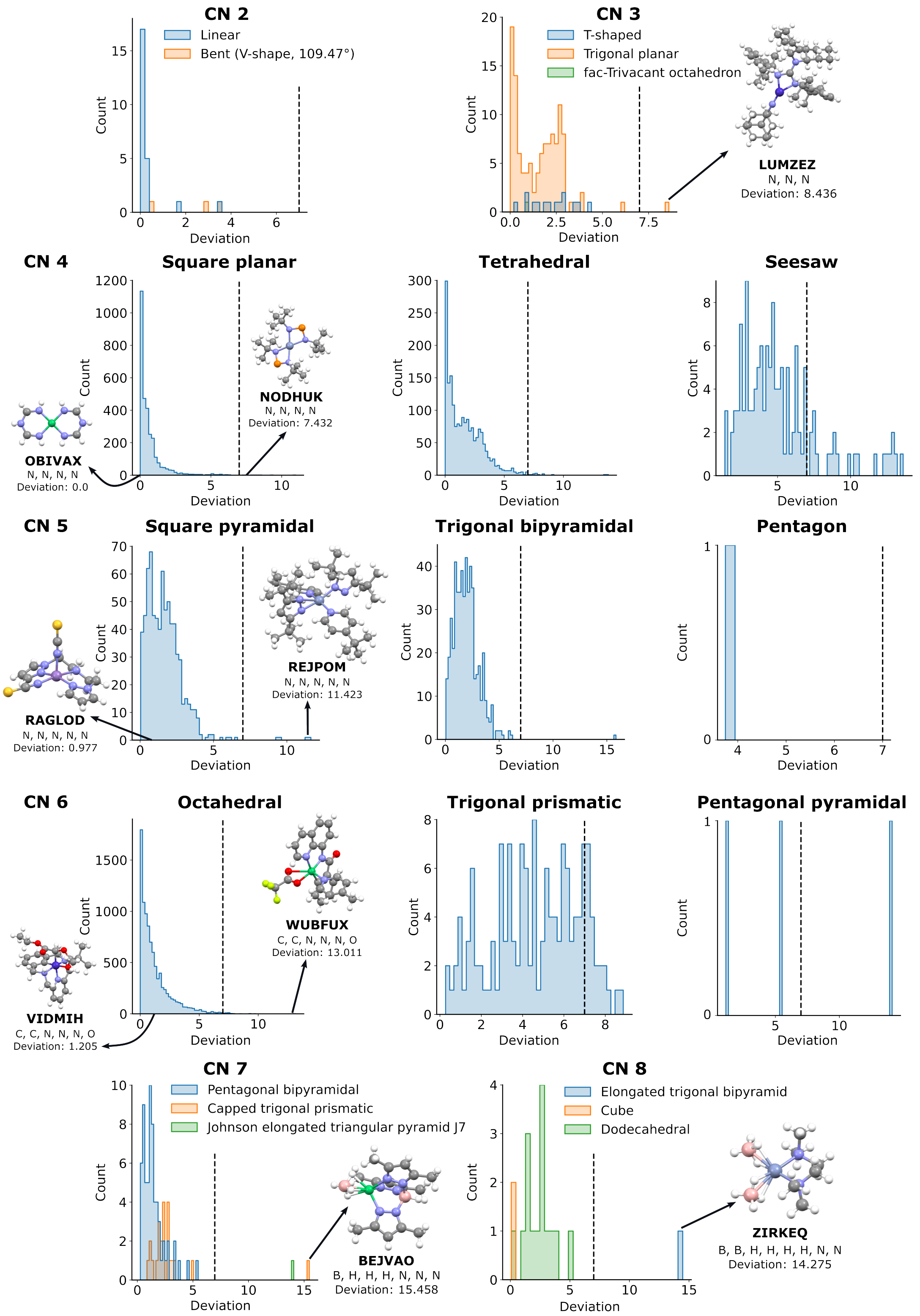

Figure S1: Distribution of deviation values from the determined coordination geometry. The coordination number (CN) ranges from 2 to 8. Vertical dashed lines represent a threshold value of 7.0, defined to exclude complexes exhibiting very significant distortions from the reference shape.

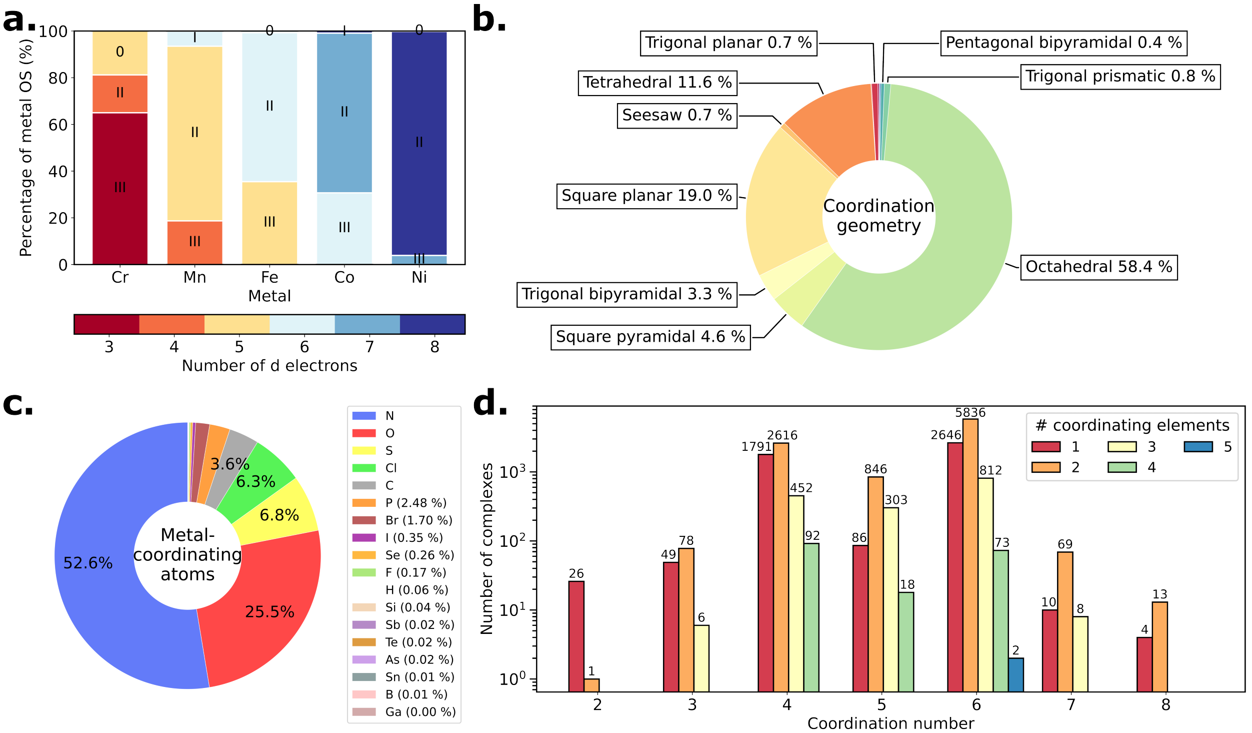

Figure S2: Chemical diversity of the original database of 15,837 complexes. **a**. Percentage of oxidation state (OS) for each metal. The OS is denoted 0, I, II, or III. The color code indicates the number of *d* electrons in the metal ion. **b**. Frequency distribution of coordination geometries. **c**. Frequency distribution of metal-coordinating atom elemental identities. **d**. Number of coordinating elements in the first coordination sphere for each coordination number.

We employed five classifiers—metal identity, oxidation state, coordination number, coordination geometry, and the composition of metal-coordinating atoms—to select 2,261 entries from 1,633 groups, categorizing the original database of 15,837 complexes. Introducing additional classifiers, *e.g.* the formula of ligands, would significantly increase the number of groups to 13,512, covering over 85 % of the original database, which would lead to a large amount of redundant complexes (see below example). We thus opted to continue using the initial five classifiers.

We illustrated the chemical diversity within a group using the example of octahedral Cr(0) complexes that coordinate six carbon atoms. This group consists of 41 complexes and can be divided into 36 subgroups based on the chemical formulas of the ligands, as detailed

in Table S1. The "Formula of Ligands" column reveals that, except for the 36th subgroup containing BIBPEI, the differences between subgroups are limited to changes in a single ligand. Consequently, we consider that sampling three complexes (highlighted in bold in Table S1) out of the 41 is sufficiently representative of the group as a whole in terms of chemical diversity.

Table S1: Subgroups in a group of octahedral Cr(0) complexes coordinating six carbon atoms based on the formula of ligands. Complexes highlighted in bold were selected for the DFT computations.

	refcode	N_{elec}	Formula of TM complex	Formula of ligands	subgroup size
1	**VOWRAA**	132	H5-C9-O6-Cr	C-O, C-O, C-O, C-O, C-O, H5-C4-O	1
2	**KOQBAT**	132	H7-C9-N-O5-Cr	C-O, C-O, C-O, C-O, C-O, H7-C4-N	1
3	**YEJWAM**	146	H6-C8-N4-O5-Cr	C-O, C-O, C-O, C-O, C-O, H6-C3-N4	1
4	HEPRID	146	H8-C11-O6-Cr	C-O, C-O, C-O, C-O, C-O, H8-C6-O	2
5	IDUMIE	150	H10-C10-O5-S-Cr	C-O, C-O, C-O, C-O, C-O, H10-C5-S	1
6	PHCBCR	150	H6-C12-O6-Cr	C-O, C-O, C-O, C-O, C-O, H6-C7-O	1
7	KARWEF	162	H10-C12-O7-Cr	C-O, C-O, C-O, C-O, C-O, H10-C7-O2	1
8	HEPREZ	162	H6-C14-O6-Cr	C-O, C-O, C-O, C-O, C-O, H6-C9-O	1
9	JOKGEV	178	H13-C13-N-O7-Cr	C-O, C-O, C-O, C-O, C-O, H13-C8-N-O2	1
10	SIMQOU	186	H14-C14-O8-Cr	C-O, C-O, C-O, C-O, C-O, H14-C9-O3	1
11	QEHQID	186	H15-C14-N-O7-Cr	C-O, C-O, C-O, C-O, C-O, H15-C9-N-O2	1
12	CHVICR	186	H17-C15-N-O6-Cr	C-O, C-O, C-O, C-O, C-O, H17-C10-N-O	1
13	EJAMUX	190	H12-C15-O8-Cr	C-O, C-O, C-O, C-O, C-O, H12-C10-O3	1
14	QESZET	190	H16-C16-N2-O5-Cr	C-O, C-O, C-O, C-O, C-O, H16-C11-N2	1
15	XALFIZ	192	H12-C18-O6-Cr	C-O, C-O, C-O, C-O, C-O, H12-C13-O	2
16	MEAMCR10	196	H14-C16-N2-O6-Cr	C-O, C-O, C-O, C-O, C-O, H14-C11-N2-O	1
17	NEXNEJ	202	H16-C19-O6-Cr	C-O, C-O, C-O, C-O, C-O, H16-C14-O	2
18	JOKLIE	202	H17-C15-N-O8-Cr	C-O, C-O, C-O, C-O, C-O, H17-C10-N-O3	1
19	PURBUC	208	H18-C17-O8-Cr	C-O, C-O, C-O, C-O, C-O, H18-C12-O3	1
20	RUJLOZ	210	H12-C21-O6-Cr	C-O, C-O, C-O, C-O, C-O, H12-C16-O	1
21	JELGAJ	220	H15-C21-N-O6-Cr	C-O, C-O, C-O, C-O, C-O, H15-C16-N-O	1
22	JUSXEA	226	H21-C21-N-O6-Cr	C-O, C-O, C-O, C-O, C-O, H21-C16-N-O	1
23	REHSEC	232	H21-C18-N-O9-Cr	C-O, C-O, C-O, C-O, C-O, H21-C13-N-O4	1
24	HODGAI	234	H21-C17-N-O10-Cr	C-O, C-O, C-O, C-O, C-O, H21-C12-N-O5	1
25	NASPAY	246	H32-C19-N3-O5-P-Cr	C-O, C-O, C-O, C-O, C-O, H32-C14-N3-P	1
26	ZUMMIG	248	H22-C19-O11-Cr	C-O, C-O, C-O, C-O, C-O, H22-C14-O6	2
27	YATDUT	254	H26-C26-O6-Cr	C-O, C-O, C-O, C-O, C-O, H26-C21-O	1
28	OGOGEW	264	H22-C27-O7-Cr	C-O, C-O, C-O, C-O, C-O, H22-C22-O2	1
29	UCEYAF	266	H31-C20-N4-O6-P-Cr	C-O, C-O, C-O, C-O, C-O, H31-C15-N4-O-P	1
30	YIYCIT	278	H23-C28-N-O7-Cr	C-O, C-O, C-O, C-O, C-O, H23-C23-N-O2	2
31	NASPIG	278	H34-C24-N3-O5-P-Cr	C-O, C-O, C-O, C-O, C-O, H34-C19-N3-P	1
32	FMSICR	280	H30-C19-O7-Si4-Cr	C-O, C-O, C-O, C-O, C-O, H30-C14-O2-Si4	1
33	LENZUZ	290	H38-B-C20-N-O5-Si4-Cr	C-O, C-O, C-O, C-O, C-O, H38-B-C15-N-Si4	1
34	PUHTEU	294	H26-C26-O11-Cr	C-O, C-O, C-O, C-O, C-O, H26-C21-O6	1
35	WODSIR	296	H28-C26-O11-Cr	C-O, C-O, C-O, C-O, C-O, H28-C21-O6	1
36	BIBPEI	444	H54-C54-N6-Cr	H9-C9-N, H9-C9-N, H9-C9-N,<br>H9-C9-N, H9-C9-N, H9-C9-N	1

S2 Ground state spin computations

The ground state spins of 2,261 complexes selected via stratified sampling (see above) were computed at the B3LYP*-D3(BJ)/def2-TZVP level. Table S2 shows accessible spin states based on the d electron configuration of the metal center. Out of 2,261 complexes, 184 complexes were removed due to convergence failures in all accessible spin states, small energy gap between possible ground state spins (Table S3), and significant spin contamination (Table S4). Significant spin contamination was defined as the expectation value of $\left\langle \hat{S}^2 \right\rangle$ that deviates more than 0.2 from the exact value of $S(S+1)$,[5] or more than 0.1 for the singlet and doublet ground states.

Finally after removing 184 of the 2,261 complexes, further inspection of the chemical names (as reported in the *.cif* files) and ligand compositions revealed that among the remaining entries, one contains radical ligands, and 13 have identical chemical compositions and nearly identical structures to others despite originating from different CSD entries. Consequently, these 14 complexes were excluded, bringing the total number of complexes in the final dataset to 2,063.

Table S2: Accessible spin states with corresponding total spin quantum number S based on d^n configuration of the metal center. LS: low-spin, IS: intermediate-spin, HS: high-spin.

	LS	IS	HS
d^3	1/2		3/2
d^4	0	1	2
d^5	1/2	3/2	5/2
d^6	0	1	2
d^7	1/2		3/2
d^8	0		1

Table S3: Excluded 35 complexes due to the small energy gap between possible ground state spins less than 5 kcal/mol. OS: metal oxidation state, N_d: the number of d electrons, E_{HS-LS}: energy difference between HS and LS state, E_{diff}: energy difference between two low-lying spin states, order: spin state ordering in an ascending order, where the left one has lower energy than right one.

	refcode	metal	OS	N_d	coordination geometry	coordinating atoms	E_{HS-LS}	E_{diff}	order
1	REPHOK	Cr	2	4	Tetrahedral	Cl, Cl, N, N	-39.88	4	HS-IS-LS
2	MAYSEK	Cr	2	4	Octahedral	N, N, N, N, N, N	-32.55	2.33	HS-IS-LS
3	AXUXEW	Cr	2	4	Octahedral	O, O, O, O, O, O	-32.9	0.4	HS-IS-LS
4	MOWDAD	Mn	2	5	Octahedral	N, N, N, O, O, O	-2.99	1.46	HS-IS-LS
5	XANMIK	Fe	2	6	Square planar	C, N, N, N	-38.09	3.25	HS-IS-LS
6	SIXQOI	Fe	2	6	Square planar	N, N, N, O	-34.76	0.04	IS-HS-LS
7	MASQAA	Fe	2	6	Square planar	N, N, S, S	-24	2.72	HS-IS-LS
8	MUWVIM	Fe	2	6	Tetrahedral	C, H, N, N	-16.26	0.09	HS-IS-LS
9	DAWKES	Fe	2	6	Square pyramidal	C, C, N, N, N	-14.86	3.23	IS-HS-LS
10	BQIFEC10	Fe	2	6	Octahedral	C, C, C, C, N, N	45.18	1.37	IS-LS-HS
11	QOWWAB	Fe	2	6	Octahedral	C, C, N, O, P, P	31.42	1.18	IS-LS-HS
12	JOQZAS	Fe	3	5	Seesaw	Cl, N, N, N	-22.57	3.76	HS-IS-LS
13	TUZTEO	Fe	3	5	Tetrahedral	N, N, N, N	-29.43	4.39	HS-IS-LS
14	ZEQQUH	Fe	3	5	Square pyramidal	Cl, N, N, N, N	-15.76	0.54	IS-HS-LS
15	CELVEU	Fe	3	5	Square pyramidal	Cl, O, O, S, S	-22.03	3.61	HS-IS-LS
16	PIBROH	Fe	3	5	Square pyramidal	F, N, N, N, N	-20.31	0.36	HS-IS-LS
17	XURKIE	Fe	3	5	Square pyramidal	F, N, N, N, N	-19.09	0.4	IS-HS-LS
18	ZAWJEM	Fe	3	5	Square pyramidal	N, N, N, N, N	-15.11	0.9	IS-HS-LS
19	SILKUT	Fe	3	5	Square pyramidal	N, N, N, N, O	-13.09	3.33	HS-IS-LS
20	IBOGIQ	Fe	3	5	Octahedral	Cl, N, N, N, N, N	-18.14	2.16	HS-IS-LS
21	POWSIE	Fe	3	5	Octahedral	Cl, N, N, N, N, O	-4.41	4.41	HS-LS-IS
22	SIBZEI	Fe	3	5	Octahedral	N, N, N, N, O, O	-4.74	4.74	HS-LS-IS
23	AXUQAN	Co	2	7	Square planar	O, O, O, O	3.6	3.6	LS-HS
24	LIYLIO	Co	2	7	Square planar	O, O, O, O	3.83	3.83	LS-HS
25	VEBHEQ	Co	2	7	Square planar	S, S, S, S	-0.79	0.79	HS-LS
26	ZUBCUW	Co	2	7	Octahedral	N, N, N, N, N, N	-2.05	2.05	HS-LS
27	NUVTOO	Co	2	7	Octahedral	N, N, N, N, O, O	-4.38	4.38	HS-LS
28	TUJPUJ	Co	3	6	Square planar	N, N, N, N	52.81	2.02	LS-IS-HS
29	NIGDIR	Co	3	6	Square planar	S, S, S, S	36.34	4.61	IS-LS-HS
30	CEXGOC	Co	3	6	Square pyramidal	N, N, O, O, O	7.14	0.11	LS-IS-HS
31	TUMYIL	Co	3	6	Trigonal bipyramidal	Cl, N, N, O, O	-14.8	4.33	IS-HS-LS
32	XUNSOO	Ni	2	8	Trigonal planar	Cl, N, N	4.66	4.66	LS-HS
33	YIZXIQ	Ni	2	8	Trigonal planar	F, N, N	-3.95	3.95	HS-LS
34	XEHHIC	Ni	2	8	Square pyramidal	N, N, O, O, O	2.63	2.63	LS-HS
35	QIWPAN	Ni	3	7	Square planar	S, S, S, S	-1.23	1.23	HS-LS

Table S4: Excluded 22 complexes due to the significant spin contamination in the ground state. These complexes exhibit an expectation value of $\left\langle \hat{S}^2 \right\rangle$ deviated more than 0.2 from the exact value of $S(S+1)$ (in case of $S = 0$ or $S = 1/2$, deviation more than 0.1). OS: metal oxidation state, N_d: the number of d electrons, E_{HS-LS}: energy difference between HS and LS state, E_{diff}: energy difference between two low-lying spin states, deviation: deviation of an expectation value of of $\left\langle \hat{S}^2 \right\rangle$ from the exact value of $S(S+1)$, S: total spin quantum number for the ground state spin of a given complex.

	refcode	metal	OS	N_d	coordination geometry	coordinating atoms	E_{HS-LS}	E_{diff}	deviation	S
1	RUDXOE	Cr	2	4	Octahedral	Cl, Cl, N, N, N, N	-25.58	10.91	0.70	1
2	GENZII	Cr	2	4	Octahedral	Cl, Cl, N, N, N, N	-25.35	13.87	0.65	1
3	MUNKUB	Cr	2	4	Octahedral	Cl, N, N, N, N, N	-27.73	8.79	0.70	1
4	LUYQUU	Cr	2	4	Octahedral	N, N, O, O, O, O	-25.74	22.25	0.36	1
5	LUYQII	Cr	2	4	Octahedral	N, N, O, O, O, O	-23.73	23.7	0.34	1
6	WUNBAL	Cr	2	4	Octahedral	N, N, O, O, O, O	-20.65	16.51	0.42	1
7	LUYQOO	Cr	2	4	Octahedral	N, N, O, O, O, O	-19.71	16.67	0.40	1
8	MIFXAC	Cr	2	4	Octahedral	N, N, O, O, O, O	-27.66	7.09	0.75	1
9	HIDGUX	Cr	2	4	Octahedral	O, O, O, O, O, O	-23.89	16.4	0.43	1
10	AGICIF	Mn	2	5	Square planar	N, N, N, N	-21.37	16.22	0.33	3/2
11	LEHSIB	Mn	2	5	Square planar	N, N, O, O	3.12	23.3	0.29	3/2
12	XILLIO	Mn	2	5	Square planar	N, N, O, O	3.74	27.88	0.21	3/2
13	QOHFAV	Mn	2	5	Octahedral	N, N, N, N, S, S	10.85	7.31	0.14	1/2
14	GICKEJ	Mn	2	5	Octahedral	N, N, N, N, S, S	10.03	7.16	0.12	1/2
15	CEPMAN	Fe	2	6	Trigonal bipyramidal	C, C, N, N, N	-20.34	7.53	0.41	2
16	COSBIX	Fe	2	6	Trigonal prismatic	Cl, Cl, N, N, N, N	-34.86	21.19	0.27	2
17	UQAFAW	Fe	3	5	Trigonal planar	C, C, N	3.59	12.4	0.21	3/2
18	BADPUT	Fe	3	5	Square pyramidal	I, N, N, N, N	33.82	26.13	0.77	1/2
19	ETINAX	Fe	3	5	Square pyramidal	I, N, N, S, S	28	16.51	0.89	1/2
20	QIJBOC	Fe	3	5	Square pyramidal	N, N, N, N, S	29.42	10.67	0.69	1/2
21	MEGDOR	Co	1	8	Octahedral	N, N, N, N, N, N	-33.79	33.79	0.44	1
22	TAHDIS	Co	2	7	Square planar	C, C, N, N	9.58	9.58	0.57	1/2

Due to the aforementioned filtering and exclusion processes, 78 groups in Table S5 were not represented in the final dataset, termed as the TM-GSspin dataset. Most of these unrepresented groups had a group size of 1 in the original database, leaving no alternatives available. For groups with more than one entry, DFT calculations were performed on one to three additional complexes. However, if these additional complexes also failed in the computations, those groups are noted in Table S5. Despite the reduction from 1,633 to 1,555 groups, the dataset still exhibits large chemical diversity (see Figure 2 in the main text).

Table S5: Unrepresented 78 groups in the TM-GSspin dataset due to the exclusion of 198 complexes out of 2,261. OS: metal oxidation state, N_d: the number of d electrons, CN: coordination number.

	metal	OS	N_d	CN	coordination geometry	coordinating atoms	group size
1	Cr	2	4	2	Linear	S, S	1
2	Cr	2	4	3	T-shaped	N, N, Si	1
3	Cr	2	4	4	Square planar	N, N, N, O	1
4	Cr	2	4	4	Tetrahedral	Cl, Cl, N, N	1
5	Cr	2	4	6	Octahedral	Cl, Cl, N, N, N, N	2
6	Cr	2	4	6	Octahedral	Cl, Cl, N, N, O, O	1
7	Cr	2	4	6	Octahedral	Cl, N, N, N, N, N	1
8	Cr	2	4	6	Octahedral	O, O, O, O, O, O	2
9	Cr	3	3	3	Trigonal planar	I, N, N	1
10	Cr	3	3	3	Trigonal planar	O, O, O	1
11	Cr	3	3	5	Square pyramidal	C, C, C, C, C	1
12	Cr	3	3	5	Square pyramidal	C, C, C, O, O	1
13	Mn	1	6	6	Octahedral	C, C, C, Te, Te, Te	1
14	Mn	2	5	2	Linear	C, C	2
15	Mn	2	5	2	Linear	S, S	1
16	Mn	2	5	3	Trigonal planar	C, C, N	1
17	Mn	2	5	3	Trigonal planar	O, O, O	1
18	Mn	2	5	4	Seesaw	Br, N, N, N	1
19	Mn	2	5	4	Seesaw	I, I, N, N	1
20	Mn	2	5	4	Square planar	N, N, N, N	1
21	Mn	2	5	4	Square planar	N, N, O, O	2
22	Mn	2	5	4	Tetrahedral	C, C, C, Si	1
23	Mn	2	5	4	Tetrahedral	C, N, N, N	1
24	Mn	2	5	4	Tetrahedral	C, N, N, O	1
25	Mn	2	5	4	Tetrahedral	Se, Se, Se, Se	1
26	Mn	2	5	5	Square pyramidal	Br, N, N, N, N	1
27	Mn	2	5	5	Square pyramidal	N, N, O, S, S	1
28	Mn	2	5	5	Trigonal bipyramidal	N, N, N, O, O	2
29	Mn	2	5	5	Trigonal bipyramidal	N, P, S, S, S	1
30	Mn	2	5	6	Octahedral	Br, N, N, N, N, O	1
31	Mn	2	5	6	Octahedral	C, C, C, P, P, P	1
32	Mn	2	5	6	Octahedral	Cl, Cl, S, S, S, S	2
33	Mn	2	5	6	Octahedral	Cl, N, N, O, O, O	1
34	Mn	2	5	6	Octahedral	I, I, O, O, O, O	1
35	Mn	2	5	6	Octahedral	N, N, N, O, S, S	1
36	Mn	2	5	6	Pentagonal pyramidal	Cl, N, N, N, N, N	1
37	Mn	2	5	7	Pentagonal bipyramidal	N, N, N, N, N, N, O	1
38	Mn	3	4	5	Trigonal bipyramidal	O, O, O, O, O	1
39	Fe	2	6	2	Bent (V-shape, 109.47°)	N, N	1

Continued on next page

Table S5 – continued from previous page

	metal	OS	N_d	CN	coordination geometry	coordinating atoms	group size
40	Fe	2	6	2	Bent (V-shape, 109.47°)	S, S	1
41	Fe	2	6	2	Linear	O, O	2
42	Fe	2	6	3	Trigonal planar	C, C, N	1
43	Fe	2	6	4	Square planar	N, N, S, S	1
44	Fe	2	6	4	Tetrahedral	C, H, N, N	1
45	Fe	2	6	5	Square pyramidal	C, C, N, N, N	2
46	Fe	2	6	5	Square pyramidal	N, N, N, N, P	1
47	Fe	2	6	5	Trigonal bipyramidal	C, C, N, N, N	3
48	Fe	2	6	6	Octahedral	C, C, N, O, P, P	1
49	Fe	3	5	3	Trigonal planar	C, C, N	1
50	Fe	3	5	4	Seesaw	Cl, N, N, N	1
51	Fe	3	5	5	Square pyramidal	Cl, O, O, S, S	1
52	Fe	3	5	5	Square pyramidal	I, N, N, S, S	2
53	Fe	3	5	5	Square pyramidal	N, N, N, N, S	4
54	Fe	3	5	6	Octahedral	N, N, N, N, P, P	1
55	Fe	3	5	6	Trigonal prismatic	N, N, N, N, O, O	1
56	Co	1	8	6	Octahedral	N, N, N, N, N, N	1
57	Co	2	7	2	Linear	S, S	1
58	Co	2	7	3	Trigonal planar	Cl, O, O	1
59	Co	2	7	4	Seesaw	N, N, N, S	1
60	Co	2	7	4	Square planar	O, O, O, O	3
61	Co	2	7	4	Tetrahedral	Br, Br, C, C	1
62	Co	2	7	4	Tetrahedral	C, C, S, S	1
63	Co	2	7	4	Tetrahedral	F, N, N, O	1
64	Co	2	7	4	Tetrahedral	I, N, N, N	1
65	Co	2	7	5	Square pyramidal	Cl, N, N, N, O	1
66	Co	2	7	5	Square pyramidal	H, S, S, S, S	1
67	Co	2	7	6	Trigonal prismatic	N, N, N, N, N, O	1
68	Co	3	6	4	Tetrahedral	C, C, C, N	1
69	Co	3	6	5	Trigonal bipyramidal	Cl, N, N, O, O	1
70	Co	3	6	6	Octahedral	As, As, As, As, Cl, P	1
71	Co	3	6	6	Octahedral	N, N, N, N, Se, Se	1
72	Ni	2	8	2	Linear	N, N	1
73	Ni	2	8	3	Trigonal planar	Cl, N, N	1
74	Ni	2	8	3	Trigonal planar	F, N, N	1
75	Ni	2	8	4	Square planar	Br, C, C, N	1
76	Ni	2	8	4	Square planar	C, O, P, S	1
77	Ni	2	8	4	Tetrahedral	I, I, P, P	1
78	Ni	2	8	4	Tetrahedral	O, P, P, P	1

The B3LYP* results were compared to TPSSh and M06L using the same def2-TZVP basis set. This comparison was conducted on a subset of 445 Fe complexes, as depicted in Figure S3. Notably, when excluding the complexes with significant spin contamination or small energy gaps between possible ground state spins, the number of low-spin (LS), intermediate-spin (IS), and high-spin (HS) complexes is consistent across all three functionals.

Figure S4 shows the ground state spins of complexes with 3d, 4d, or 5d transition metal (TM) ions, which were determined at the B3LYP*-D3(BJ)/def2-TZVP level. As expected, the ground state spins of 3d coordination complexes with 0, 1, 2, 3, 9, or 10, *d* electrons are determined based on the number of unpaired electrons. Some 3d complexes containing haptic ligands with 2, 3 or 4 *d* electrons exhibit the LS state while 3d coordination complexes with *d* electron numbers of 2, 3, or 4 display triplet or quartet ground states. On the other hand, the ground state spins of 4d or 5d complexes are typically LS state. In the case of heavy metal complexes with haptic ligands, their ground state spins are predominantly in the LS state.

We also conducted additional computations to determine the ground state spins of nitrosyl complexes, which were originally excluded at the dataset curation process. Generally, the ground state spins of nitrosyl complexes favor the LS states (Table S6). *cell2mol* has assigned the ligand charge of 0 to linear nitrosyl and -1 to bent one based on the metal–nitrogen–oxygen angle. However, in specific cases (highlighted in red in Table S6), we observed a mismatch between the ground state spin of the complex and the possible spin states of the metal ion. This discrepancy arises from a spin originating from a nitrosyl ligand, which was assigned to a linear type by *cell2mol* but appears to be a bent type.

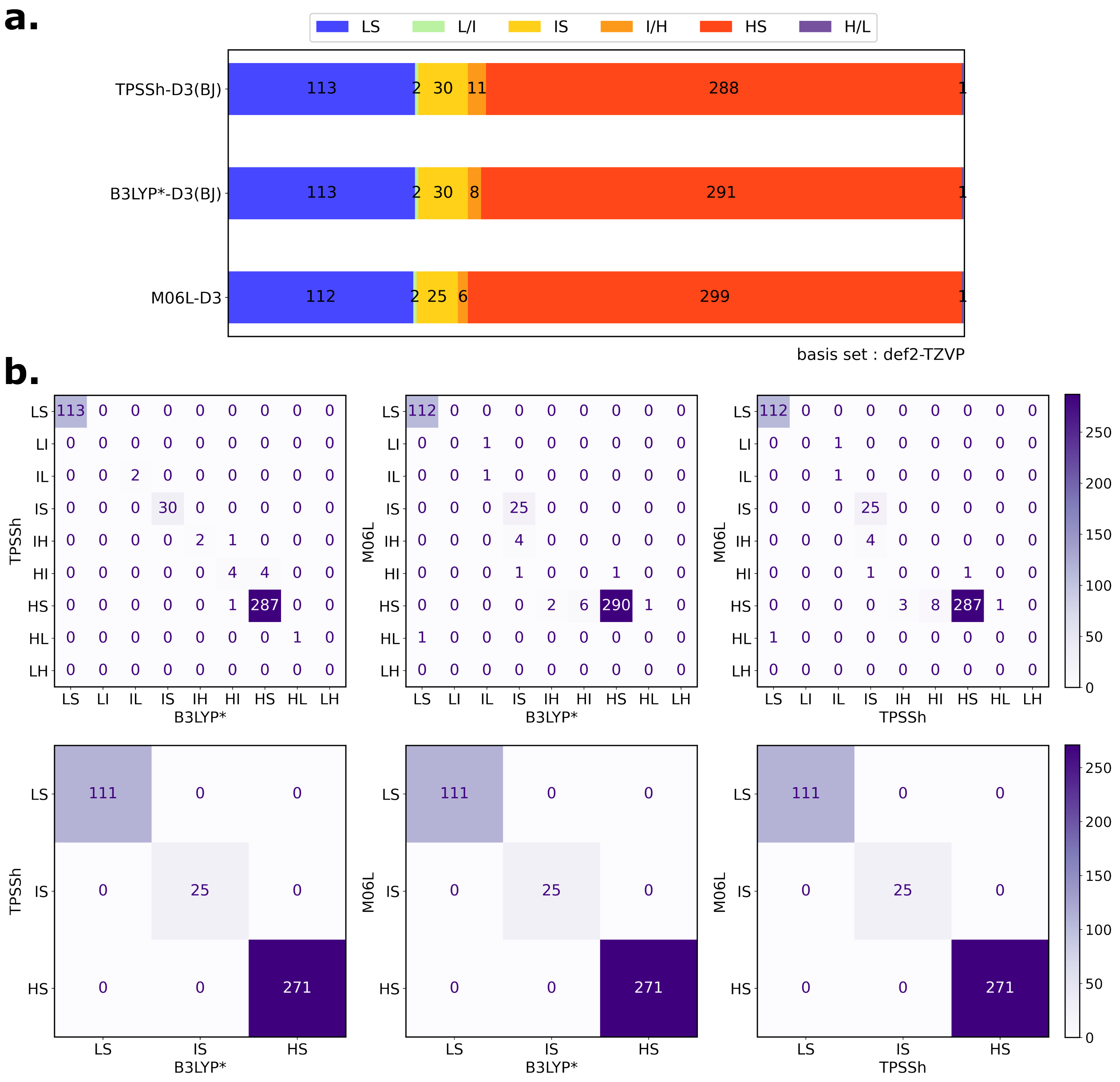

Figure S3: Benchmark of DFT functionals B3LYP*, TPSSh, and M06L. **a.** Comparison of ground state spins in 445 Fe complexes, computed with B3LYP*, TPSSh, and M06L using the def2-TZVP basis set. LS: low-spin, IS: intermediate-spin, HS: high-spin. L/I, I/H, and H/L denote complexes with energy gaps less than 5 kcal/mol between two low-lying spin states. **b.** Confusion matrices before (top) or after (bottom) filtering complexes with significant spin contamination or small energy gaps. In top panels, complexes with small energy gaps represent as LI (LS $<$ IS), IL (IS $<$ LS), IH (IS $<$ HS), HI (HS $<$ IS), HL (HS $<$ LS), or LH (LS $<$ HS).

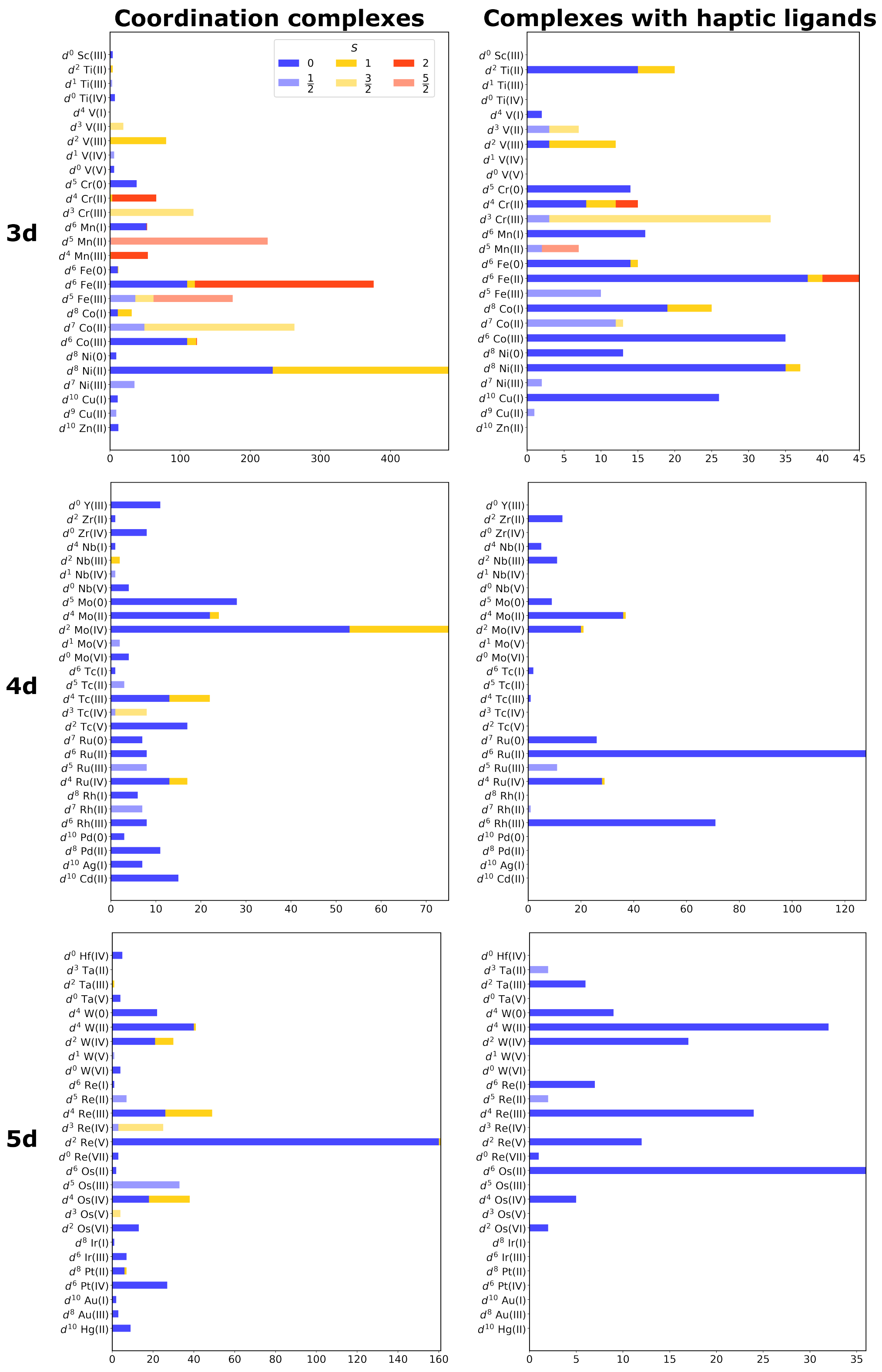

Figure S4: Ground state spins of mononuclear TM complexes with 3d (top), 4d (middle), or 5d (bottom) metal center. The left panels show the ground state spins of coordination complexes, and the right panels show those of complexes with haptic ligands.

Table S6: Ground state spin of nitrosyl complexes initially excluded during the dataset curation process. OS: metal oxidation state, N_d: the number of d electrons, N_{NO}: the number of nitrosyl ligands present in the complex, E_{HS-LS}: energy difference between HS and LS state, E_{diff}: energy difference between two low-lying spin states, deviation: deviation of an expectation value of $\left\langle \hat{S}^2 \right\rangle$ from the exact value of $S(S+1)$, S: total spin quantum number for the ground state spin of each complex. The complexes highlighted in red show a discrepancy between the ground state spin of the complex and the possible spin states of the metal ion due to a spin originating from the nitrosyl ligand. The complexes colored in blue represent cases where E_{diff} is less than 5 kcal/mol or where the computation failed to converge for one of the accessible spin states.

	refcode	metal	OS	N_d	coordination geometry	N_{NO}	type	E_{HS-LS}	E_{diff}	deviation	S
1	JIZNAH	Cr	2	4	Octahedral	2	Linear	68.94	28.93	0	0
2	XOTXEK	Cr	2	4	Octahedral	1	Linear	70.19	38	0.58	1/2
3	SOLTUL	Mn	1	6	Tetrahedral	4	Linear	79.48	43.45	0	0
4	FIZQEL	Mn	2	5	Octahedral	1	Linear	59.63	22.45	0	0
5	WOPGAK	Mn	3	4	Square pyramidal	1	Linear	61.27	24.65	0.31	1/2
6	ZOXKOO	Fe	2	6	Trigonal bipyramidal	2	Linear	73.92	40.71	0	0
7	LUGYOB	Fe	2	6	Octahedral	1	Bent	-12.4	17.47	0.07	1
8	DAQSUM	Fe	2	6	Octahedral	1	Linear	15.97	17.75	0.78	3/2
9	CEMNOZ	Fe	3	5	Seesaw	2	Linear	61.51	29.32	1.45	1/2
10	XUQTOU	Fe	3	5	Tetrahedral	2	Linear	48.53	2.2		–
11	VEGFIX	Fe	3	5	Square pyramidal	1	Linear	–	12.24		–
12	VIVNIZ	Fe	3	5	Square pyramidal	1	Linear	54.64	24.53	0	0
13	PIMVOZ	Fe	3	5	Square pyramidal	1	Linear	56.67	21.28	0	0
14	VIVNEV	Fe	3	5	Square pyramidal	1	Linear	50.92	12.03	0	0
15	VEGFET	Fe	3	5	Square pyramidal	1	Linear	52.48	12.53	0	0
16	VETXUN	Fe	3	5	Square pyramidal	1	Linear	24.1	8.86	0	0
17	VUPTUX	Fe	3	5	Square pyramidal	2	Bent	63.74	28.91	1.12	1/2
18	SURHAQ	Fe	3	5	Square pyramidal	1	Bent	45.08	35.72	0.26	1/2
19	VUPTOR	Fe	3	5	Square pyramidal	2	Bent	58.87	34.63	1.28	1/2
20	RAWHAZ	Fe	3	5	Square pyramidal	1	Bent	44.25	27.37	0.45	1/2
21	WUCCAY	Fe	3	5	Square pyramidal	1	Linear	49.3	15.96	0	0
22	ABIJEB	Fe	3	5	Square pyramidal	1	Bent	44.34	16.71	0.41	1/2
23	NELBIP	Fe	3	5	Octahedral	1	Linear	107.13	36.46	0	**0**
24	HADZAO	Fe	3	5	Octahedral	1	Linear	105.91	36.81	0	0
25	MOTCIJ	Fe	3	5	Octahedral	1	Bent	80.93	33.25	0.06	1/2
26	LIWNAH	Fe	3	5	Octahedral	1	Linear	56.63	30.09	0	0
27	BERJIS	Fe	3	5	Octahedral	1	Linear	51.59	18.63	0	0
28	YOYMIJ	Fe	3	5	Octahedral	2	Bent	56.35	32.86	1.41	1/2
29	QORFIO	Fe	3	5	Octahedral	1	Linear	64.24	32.78	0	0
30	XIFBIX	Fe	3	5	Octahedral	1	Linear	48.95	24.45	0	0
31	NSACCO	Co	1	8	Tetrahedral	2	Linear	44.57	44.57	0	0
32	OCAHOP	Co	3	6	Square pyramidal	1	Bent	60.78	20.93	0	0
33	CEFTEM	Co	3	6	Square pyramidal	1	Bent	45.22	22.4	0	0
34	VIZZAG	Co	3	6	Square pyramidal	1	Bent	40.04	15.37	0	0

To evaluate the impact of geometry optimization, we compared the effect of four different geometry optimization strategies in 10 exemplary complexes (see Table S7): no optimization of the crystallographic structure, optimization of only hydrogen atoms, optimization of all atoms except the metal and its coordinating atoms, and full geometry optimization.

Table S7: Exemplary complexes to assess the impact of geometry optimization on spin-splitting energy. OS: metal oxidation state, N_d: the number of d electrons, spin multiplicity in the ground state.

	refcode	metal	OS	N_d	coordination geometry	coordinating atoms	total charge	spin multiplicity
1	PUVLAV	Cr	2	4	Seesaw	Cl, Cl, O, O	0	5
2	SCYSCR	Cr	3	3	Octahedral	N, N, O, O, S, S	-1	4
3	WIFSEJ	Mn	2	5	Octahedral	O, O, O, O, S, S	0	6
4	JEDJAE	Mn	3	4	Octahedral	O, O, O, O, O, O	-5	5
5	XABHIV	Fe	2	6	Octahedral	C, C, C, H, P, P	1	1
6	KAJTOE	Fe	3	5	Trigonal bipyramidal	Cl, Cl, Cl, O, O	0	6
7	METGOH	Co	2	7	Tetrahedral	Cl, Cl, N, N	0	4
8	PEXKAF	Co	3	6	Octahedral	Cl, N, N, N, N, O	2	1
9	HUBLEW	Ni	2	8	Square planar	N, S, S, S	-1	1
10	TAGFIR	Ni	3	7	Square planar	N, N, S, S	-1	2

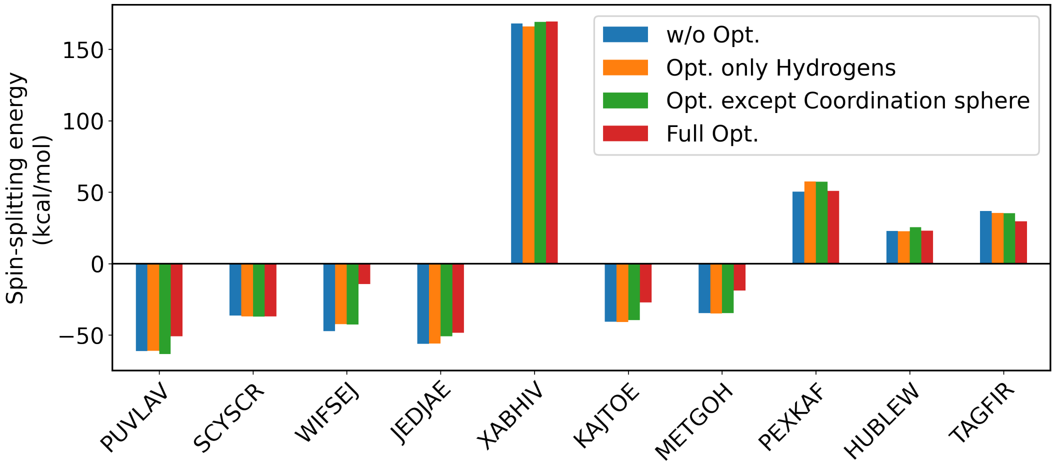

Figure S5: Impact of geometry optimization on spin-splitting energy in 10 exemplary complexes. No optimization (blue), optimization performed only for hydrogen atoms (orange), optimization performed on all atoms except the metal and its coordinating atoms (green), full geometry optimization for all atoms (red).

Figure S5 shows that the effects of geometry optimization on spin-splitting energies are minor, and the resulting ground state spins remain unchanged. Since we do not consider spin crossover complexes in our dataset, which should be the systems where the spin state is most sensitive to geometry, we believe that the effect of crystal packing and geometry relaxation is minimal.

In the final TM-GS spin dataset, 32 complexes have hydrogens as coordinating atoms. Geometry optimization for hydrogens in the LS state completed successfully for all these complexes, except for one Co(II) tetrahedral complex, which was optimized in the HS state instead. Final ground state spins were determined through single-point energy computations for different spin states, revealing 27 LS and 5 HS complexes. For the four complexes in the HS ground state, geometry optimization only for hydrogens in the LS state presented no issues.

Lastly, following iterative and automated corrections were applied in case of convergence failures:

- We automatically turned on integral symmetry in the SCF if the run terminated due to no symmetry.
- We loosened the geometry convergence threshold if the geometry optimization of the hydrogen atoms did not converge.
- If the optimization failed due to the lack of a lower energy point, we automatically tried other spin states. For example, the square pyramidal Ni(II) complex (CSD refcode: TITRAQ) did not converge in the singlet state, so we refined the geometry in the triplet state, which then terminated normally. This correction happened only for 31 complexes.

S3 TM-GSspin dataset

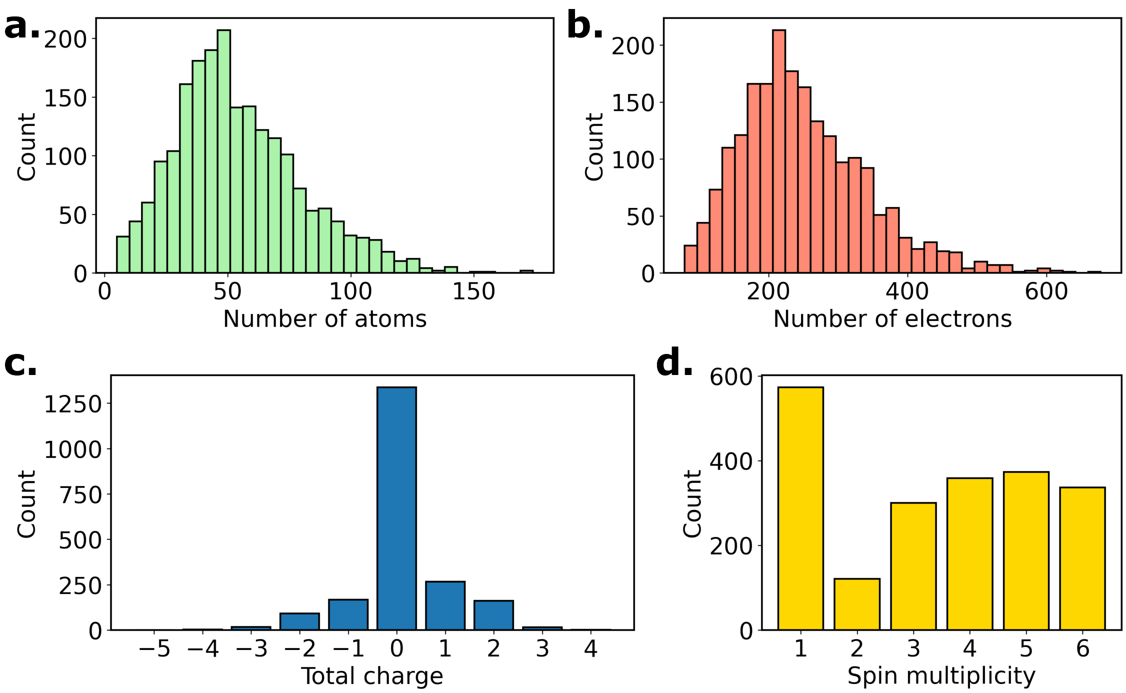

Figure S6: An overview of TM-GSspin dataset. **a.** The number of atoms, **b.** number of electrons, **c.** total molecular charges, and **d.** spin multiplicity.

We found 6,631 different ligands in the TM-GSspin dataset, including duplicates (the number of unique ligands based on chemical formula is 1,418). Figure S7 shows the number of atoms for each of the 6,631 ligands and a red dashed line indicates the average value of 16.6.

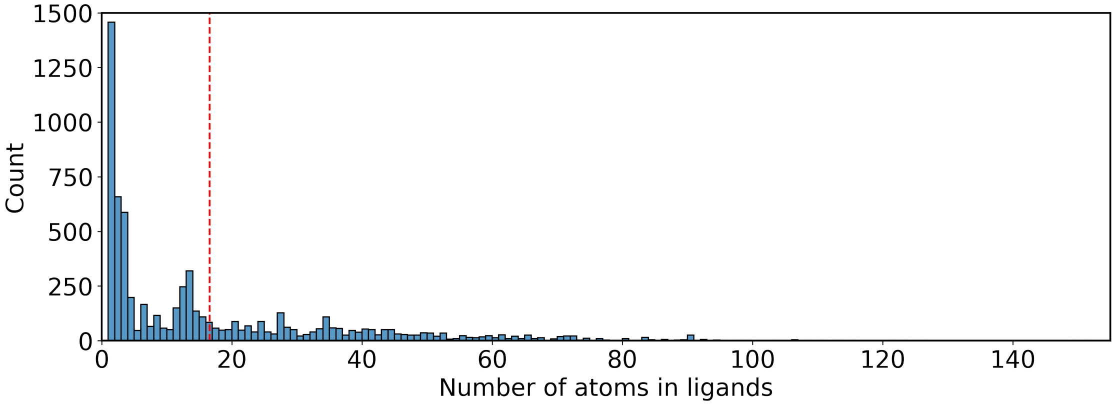

Figure S7: Number of atoms in the 6,631 ligands in TM-GSspin dataset.

Figures S8, S9, S10, S11, S12, and S13 represent the TM-GSspin dataset broken down into each metal, OS, and coordination geometry. Each column represents a total spin quantum number S, and each row represents a coordination geometry. Red horizontal lines classify coordination geometries by their coordination numbers. The number within each grid cell indicates the number of corresponding complexes in that cell. The color code represents the proportion of spin states within a given coordination geometry, where navy blue corresponds to 0 % and yellow to 100 %.

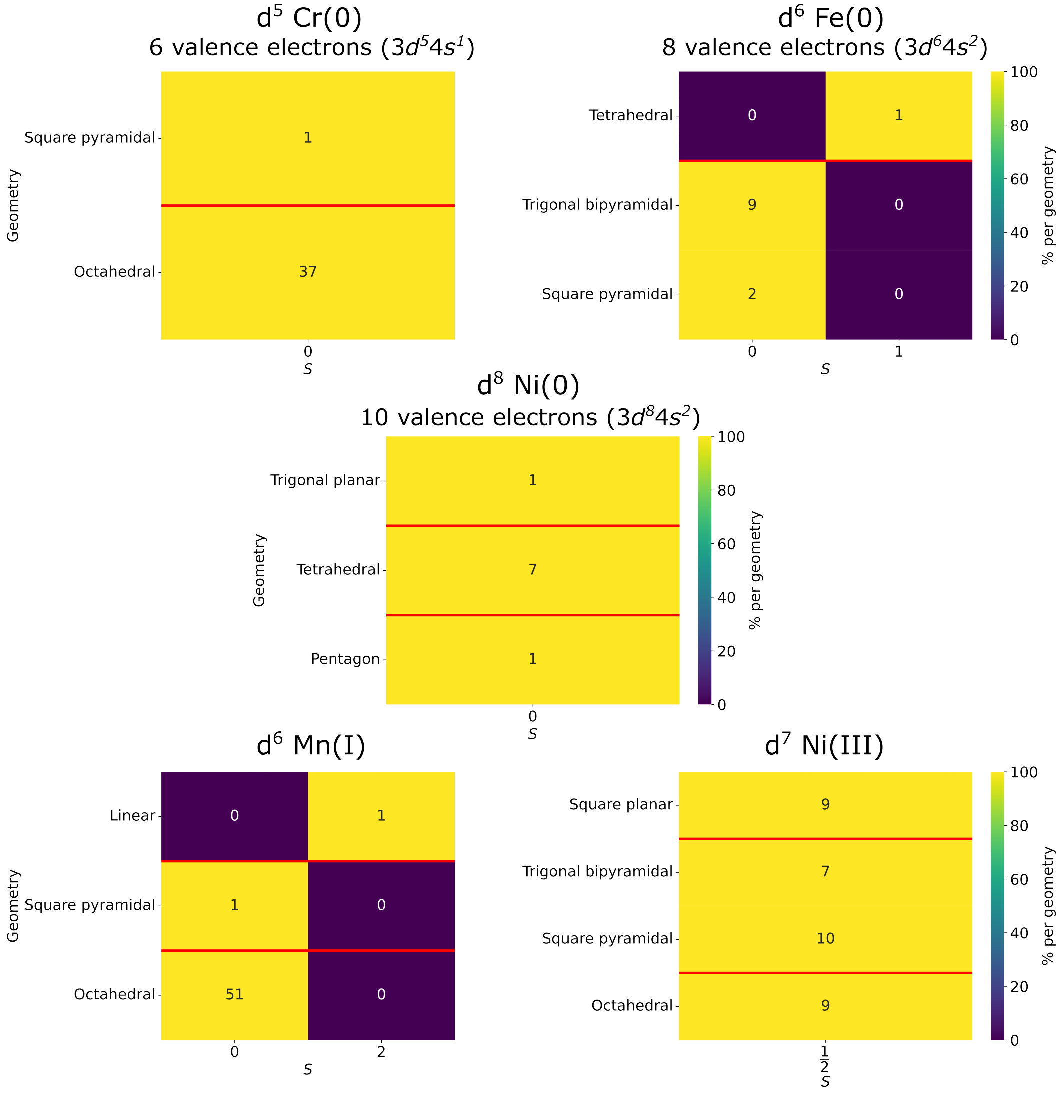

Figure S8: Ground state spin of zero-valent complexes for Cr(0), Fe(0), and Ni(0), along with d^6 Mn(I) and d^7 Ni(III) complexes.

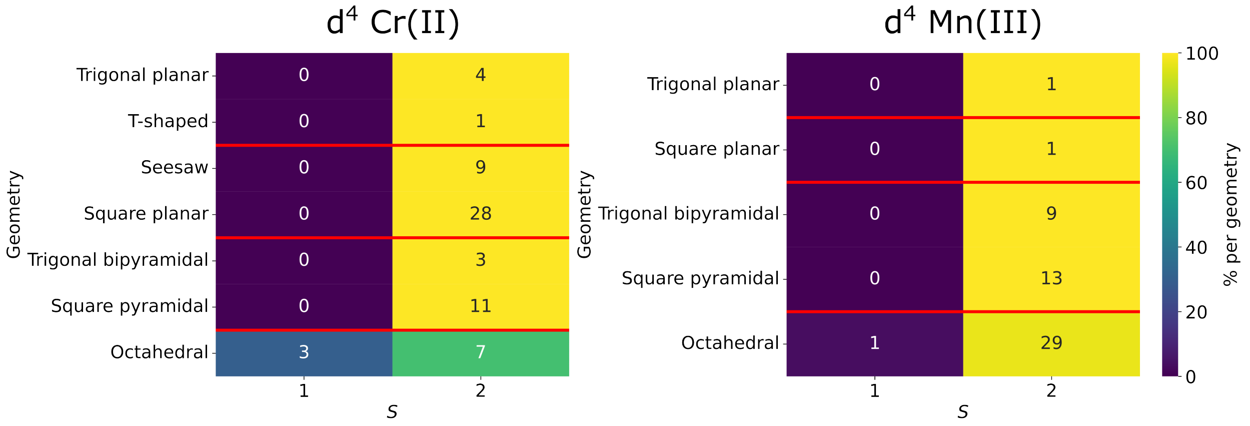

Figure S9: Ground state spin of d^4 Cr(II) and Mn(III) complexes.

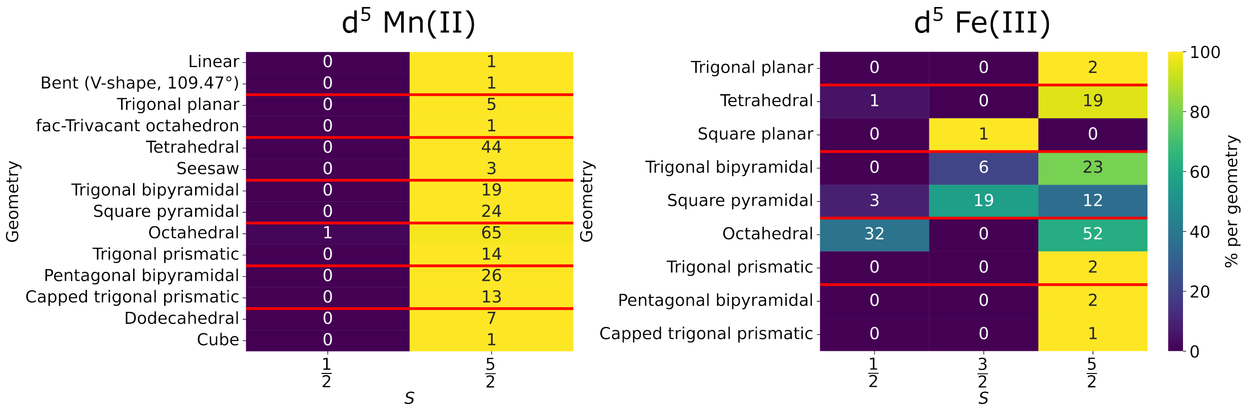

Figure S10: Ground state spin of d^5 Mn(II) and Fe(III) complexes.

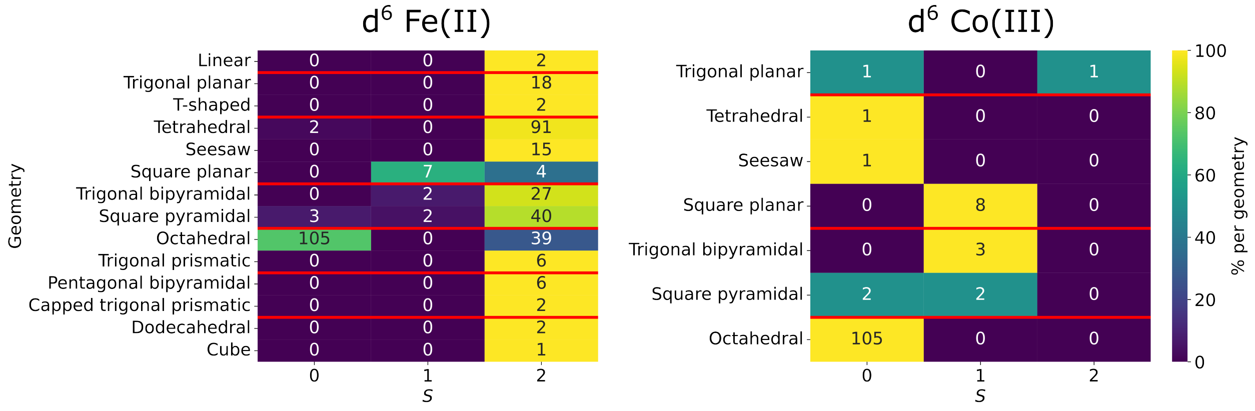

Figure S11: Ground state spin of d^6 Fe(II) and Co(III) complexes.

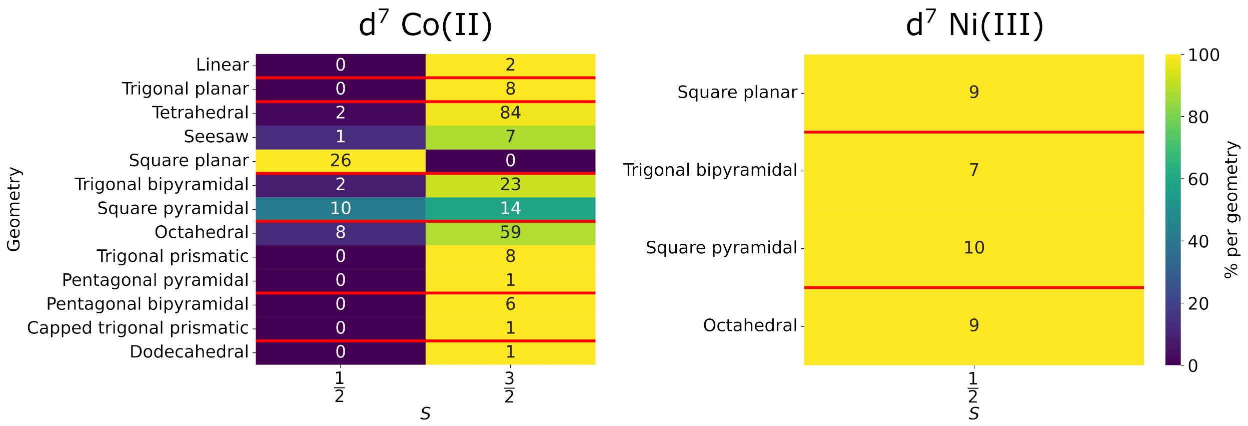

Figure S12: Ground state spin of d^7 Co(II) and Ni(III) complexes.

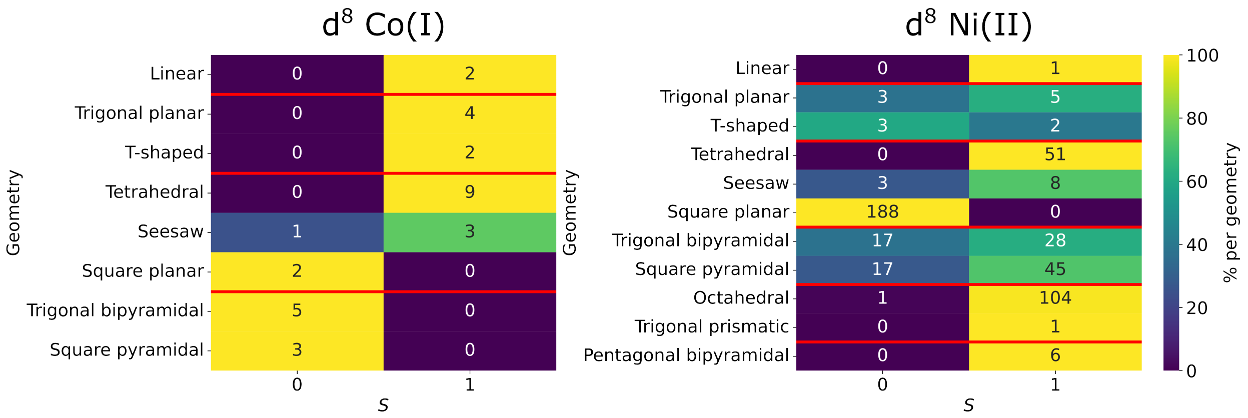

Figure S13: Ground state spin of d^8 Co(I) and Ni(II) complexes.

Figure S14 shows the ground state spins of 144 Fe(II) octahedral complexes depending on coordination sphere composition. Complexes where the major coordinating element is carbon (C) or phosphorus (P)—both strong field ligands—predominantly exhibit a LS state. In contrast, complexes where the major coordinating elements are halogens or oxygen (as shown in Figure S14a: Cl6, O6) or a combination of weak field ligands (as shown in Figure S14c: Cl4-O2, O4-Br2, O4-Cl2) generally adopt a HS state. Complexes where nitrogen (N) or sulfur (S) dominate the coordination sphere display mixed ground state spins.

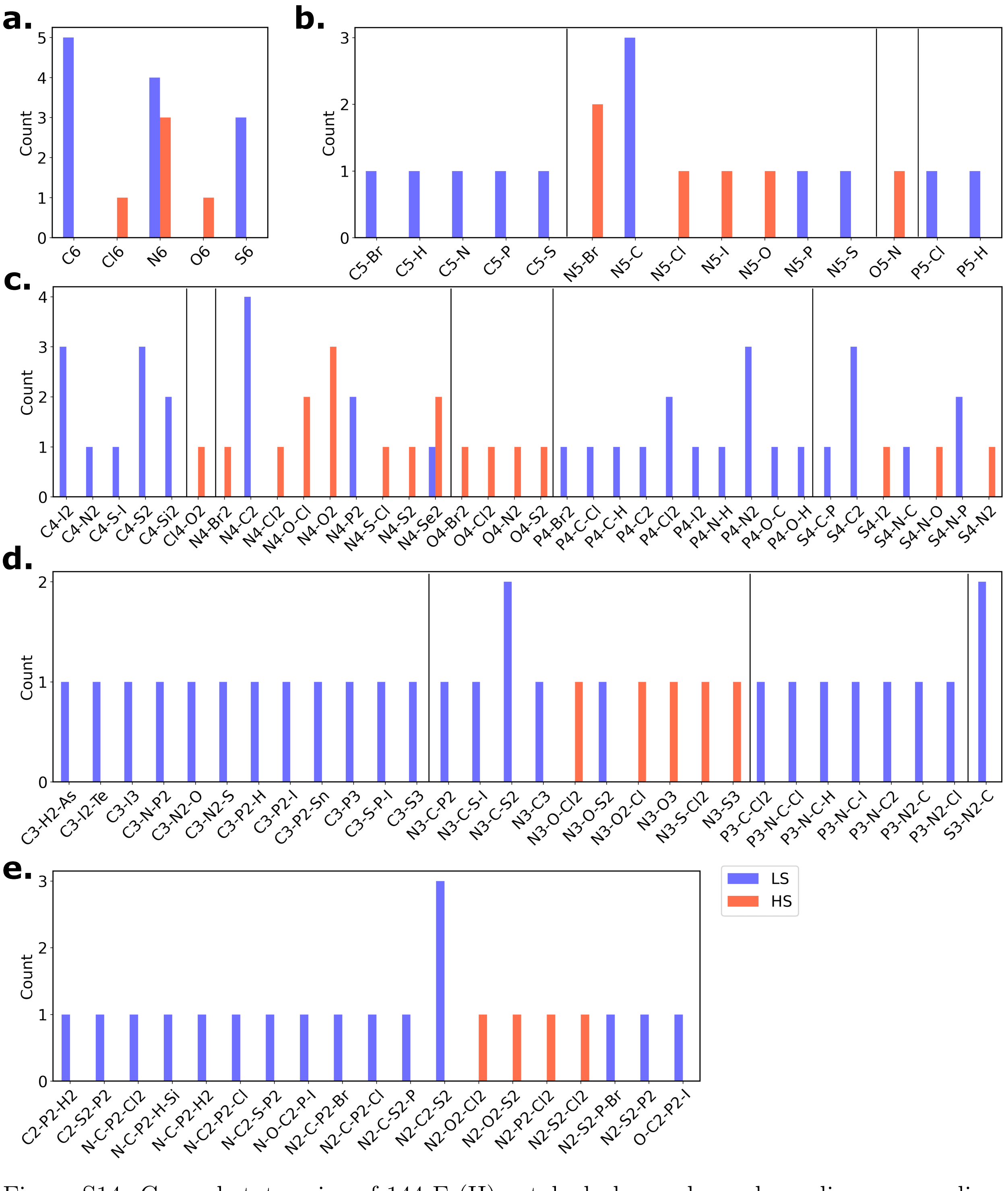

Figure S14: Ground state spins of 144 Fe(II) octahedral complexes depending on coordination sphere composition. **a.** All six coordinating atoms are identical. **b.** Five out of six coordinating atoms are the same element. **c.** Four out of six coordinating atoms are the same element. **d.** Three out of six coordinating atoms are the same element. **e.** Other coordination sphere compositions. Vertical lines demarcate figures according to the major coordinating element.

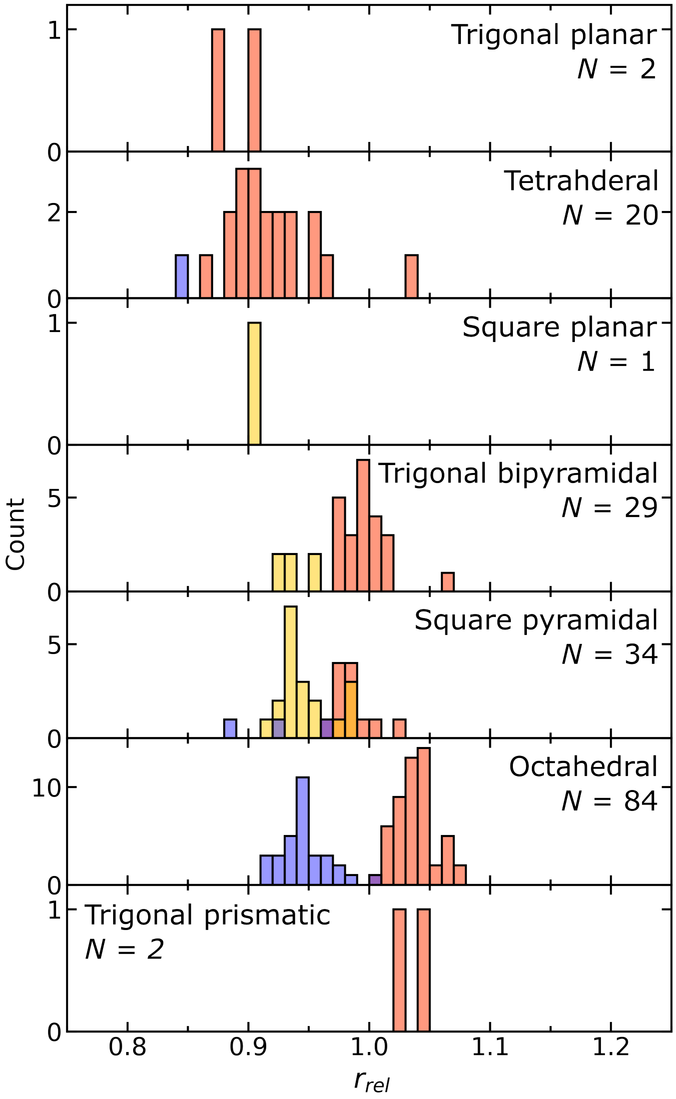

Figure S15: Histograms of relative metal radii (r_{rel}) and corresponding ground state spins for Fe(III) complexes with coordination numbers ranging from 3 to 6. N indicates the number of complexes used to plot each histogram. The color code represents the ground state spin: blue (singlet), yellow (triplet), and red (quintet).

S4 Ground state spin assignment based on empirical rules

Table S8: Covalent radii used in this work. These values are sourced from a previous analysis of experimental crystal structures.[6] For metal elements, proposed covalent radii are based on the analysis of the complexes with coordination number 6. For Mn, Fe, and Co, we use the proposed covalent radii in the LS state.

Element	Covalent radius (Å)
Cr	1.39
Mn	1.39
Fe	1.32
Co	1.26
Ni	1.24
As	1.19
B	0.84
Br	1.20
C	0.73
Cl	1.02
F	0.57
Ga	1.22
H	0.31
I	1.39
N	0.71
O	0.66
P	1.07
S	1.05
Sb	1.39
Se	1.20

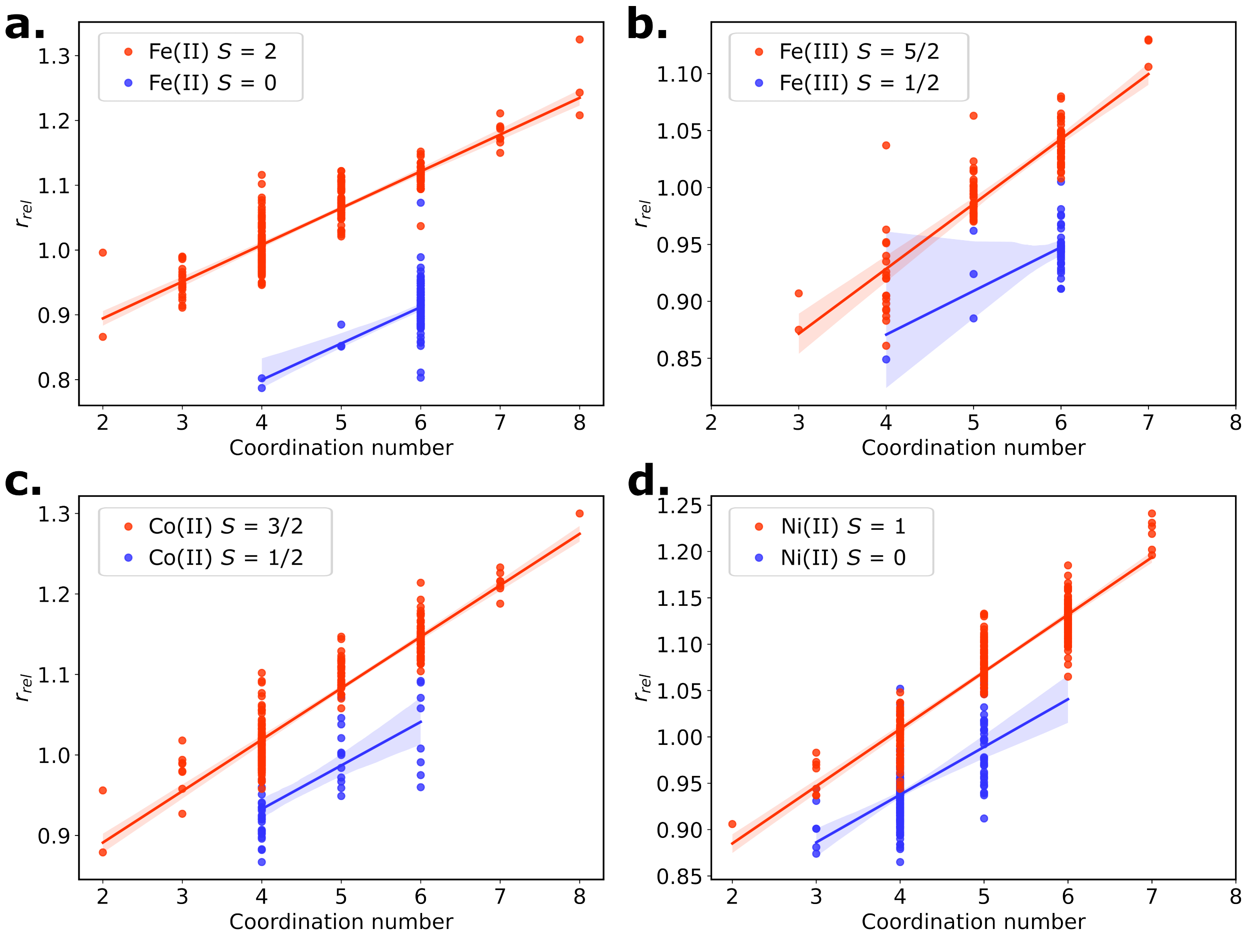

Figure S16: Relative metal radius (r_{rel}) depending on the coordination number. **a.** d^6 Fe(II) **b.** d^5 Fe(III) **c.** d^7 Co(II) **d.** d^8 Ni(II) complexes.

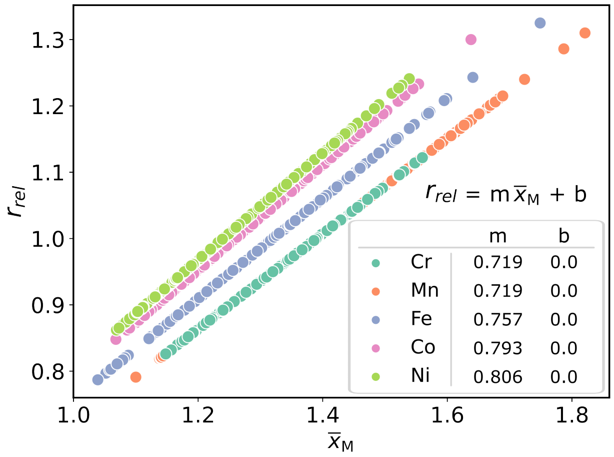

Figure S17: Relationship between relative metal radius (r_{rel}) and average of metal radius ($\bar{x}_{\mathrm{M}}$) for different metal centers. $\bar{x}_{\mathrm{M}} = \frac{\sum_{i=1}^{CN} d(\mathrm{M{-}A}_i) - r_{\mathrm{A}_i}}{CN}$

Table S9 shows 2-coordinate complexes in TM-GSspin and their ligand information. We additionally searched the CSD for 2-coordinate mononuclear complexes with Cr, Mn, Fe, Co, or Ni. These complexes typically feature monodentate bulky ligands. This observation supports our hypothesis that 2-coordinate complexes with d electrons between 4 and 8 (excluding zero-valent complexes) generally possess bulky ligands, resulting in a weak ligand field and a HS ground state. We also identified a few Cu complexes with two halogen ligands, such as the Cu(I)Br_2 complex (CSD refcode: MIYWIE). Due to its d^{10} electron configuration, a rule-based decision tree assigns its ground state spin as a singlet, regardless of the bulkiness of the ligands. We did not find any examples of these with Cr, Mn, Fe, Co, or Ni.

Table S9: 2-coordinate complexes in TM-GSspin and their ligand information.

refcode	N_{atoms}	$N_{ligands}$	avg.N_{atoms} of ligands	N_{atoms} of ligands	formula of ligands
CUJSAD	81	2	40	40, 40	H27-C10-Si3, H27-C10-Si3
WUGCAD	103	2	51	51, 51	H26-C24-N, H26-C24-N
KAGJOU	87	2	43	43, 43	H26-C15-N-Si, H26-C15-N-Si
QIVYEZ	81	2	40	40, 40	H27-C10-Si3, H27-C10-Si3
FEGVOE	87	2	43	43, 43	H26-C15-N-Si, H26-C15-N-Si
NURPUN	115	2	57	57, 57	H32-C23-N2, H32-C23-N2
KAGHOS	55	2	27	27, 27	H18-C6-N-Si2, H18-C6-N-Si2
CURVOB	95	2	47	27, 67	H18-C6-N-Si2, H37-C30
PIVHUZ	87	2	43	43, 43	H26-C15-N-Si, H26-C15-N-Si
JARKIX	173	2	86	86, 86	H49-C36-S, H49-C36-S

Table S10: Cut-off values based on the combination of the coordination geometry and the TM element. The cut-off values of Fe complexes are bold because we compute the cut-off values of other TM complexes by multiplying a factor f. The factor is defined as ratio of slope in Figure S17. For Cr or Mn, $f = 0.719/0.757 = 0.95$. For Co, $f = 0.793/0.757 = 1.05$. For Ni, $f = 0.806/0.757 = 1.06$. The cut-off values of Fe complexes were chosen based on the distribution of relative metal radii and corresponding ground state spins of Fe(II) (Figure 3c in the main text) and Fe(III) (Figure S15), to minimize incorrect spin state assignments.

	Cut-off value				
CN & coordination geometry	Cr	Mn	**Fe**	Co	Ni
CN $<$ 4 or Tetrahedral	0.81	0.81	**0.85**	0.89	0.91
CN $=$ 4, but not Tetrahedral or Square planar	0.85	0.85	**0.90**	0.94	0.96
Square planar or CN $=$ 5	0.90	0.90	**0.95**	1.00	1.01
CN $\geq$ 6	0.95	0.95	**1.00**	1.05	1.06

S5 Ground state spin prediction with statistical models

Random forest models are trained and tested using the Python script https://github.com/lcmd-epfl/cell2mol/blob/dev/cell2mol/random_forest.py. This script conducts hyperparameter optimization by testing various combinations of the following parameters:

- n_estimators: Number of trees in the forest, with tested values [100, 200, 300].
- max_features: The fraction of features to consider when looking for the best split, with tested values [0.25, 0.5, 0.75].
- bootstrap: Whether bootstrap samples are used when building trees, with options [True, False].

To ensure reproducibility in training and testing models, we set a fixed random state of 42 for the randomized search on hyperparameters and stratified K-Fold cross-validation.

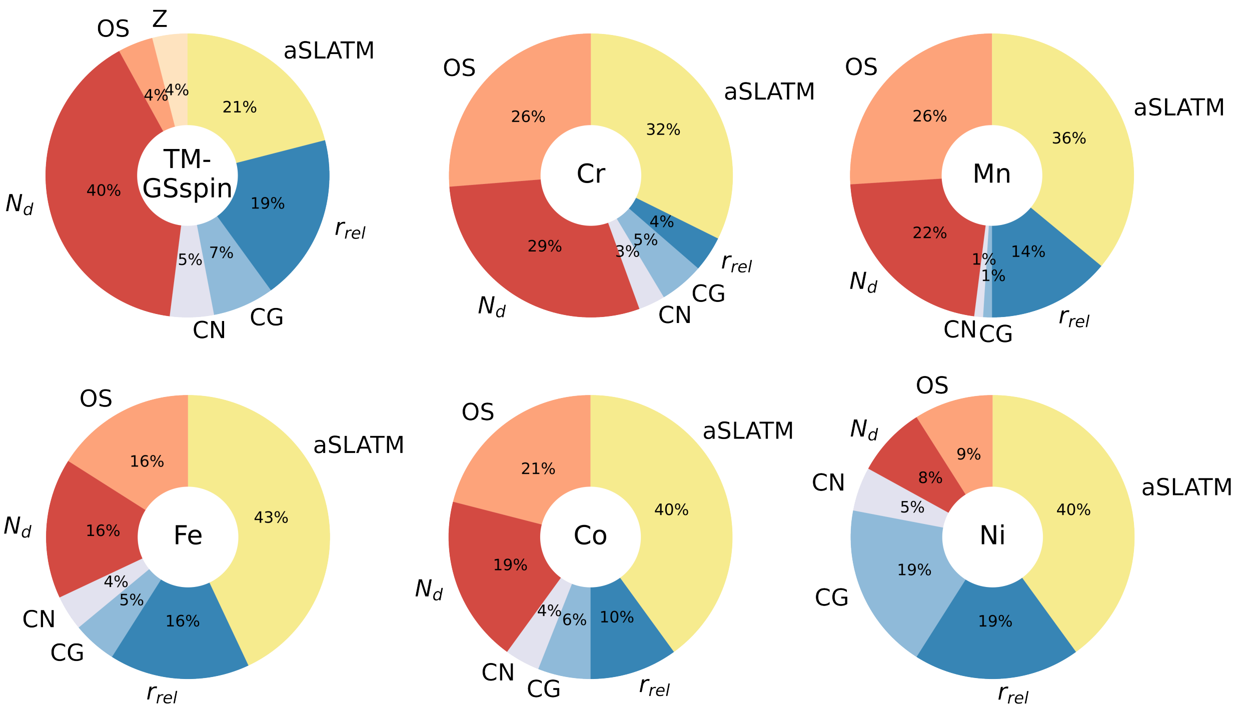

Figure S18: Feature importances in prediction models trained using $F_{\mathrm{TM+CE+aSLATM}}$. aSLATM indicates the sum of all feature importances within aSLATM.

Figure S18 shows the feature importances in models trained using $F_{\mathrm{TM+CE+aSLATM}}$. In the models trained on each metal subset, features that constitute more than 1 % within aSLATM primarily arise from two-body or three-body terms involving the central metal and one or two coordinating elements, such as carbon, nitrogen, oxygen, or phosphorus. On the other hand, in the model trained on the whole dataset, features representing more than 1 % within aSLATM predominantly stem from one-body terms associated with the different metal centers.

Table S11: Comparison of performances of random forest models using F_{CE} and F_{CE} plus the average number of atoms in ligands. 10-fold cross-validated accuracies and standard deviations (SD) are shown for the TM-GSspin dataset and for each metal subset.

Dataset	F_{CE}		F_{CE} + avg.N_{atoms} of ligands	
	Accuracy (%)	Accuracy SD (%)	Accuracy (%)	Accuracy SD (%)
TM-GSspin	46.10	2.72	51.77	3.05
Cr	91.11	5.84	92.87	4.04
Mn	97.30	2.09	97.30	2.09
Fe	86.68	3.99	88.29	2.73
Co	86.12	4.38	85.66	3.34
Ni	90.71	1.76	91.29	3.26

Table S12: Comparison of performances of random forest models using aSLATM and reduced aSLATM using principal component analysis (PCA) to reduce the number of features to 100. 10-fold cross-validated accuracies and standard deviations (SD) are shown for the TM-GSspin dataset and for each metal subset.

Dataset	aSLATM		PCA-aSLATM	
	Accuracy (%)	Accuracy SD (%)	Accuracy (%)	Accuracy SD (%)
TM-GSspin	85.26	1.00	81.68	1.93
Cr	82.04	6.48	78.44	9.29
Mn	95.51	3.30	93.69	3.39
Fe	76.19	2.93	74.61	4.13
Co	82.52	4.63	76.56	5.49
Ni	89.96	2.90	89.39	3.36

Table S13: Performance of random forest models trained on the TM-GSspin dataset and each metal subset for ground state spin prediction. Stratified 10-fold cross-validations were performed, where accuracy and F1 score indicate the mean accuracy and the mean weighted-averaged F1 score across the 10 validation sets in each dataset, respectively. SD is the mean standard deviation across the 10 validation sets in each dataset. Five different descriptors served as input features for the random forest models. F_{TM}: a vector containing the metal atomic number, metal OS, and the number of d electrons, F_{CE}: a vector containing coordination number, coordination geometry, and relative metal radius, $F_{\mathrm{TM+CE}}$: a vector incorporating F_{TM} and F_{CE}, aSLATM: atomic SLATM representation[7] of the metal center, calculated using the QML package[8] with a modified discretization grid (0.4 a.u.) and cutoff (4.0 a.u.), $F_{\mathrm{TM+CE+aSLATM}}$: a vector incorporating $F_{\mathrm{TM+CE}}$ and aSLATM. The number of complexes within each dataset is shown in parentheses. To ensure reproducibility in training and testing the random forest models, we set a fixed random state for the randomized search on hyperparameters and stratified K-Fold cross-validation.

Dataset	Descriptor	Accuracy (%)	Accuracy SD (%)	F1 score	F1 score SD
TM-GS spin (2063)	F_{TM}	75.96	1.92	0.73	0.02
	F_{CE}	46.10	2.72	0.45	0.03
	$F_{\mathrm{TM+CE}}$	97.92	0.72	0.98	0.01
	aSLATM	85.26	1.00	0.85	0.01
	$F_{\mathrm{TM+CE+aSLATM}}$	96.41	1.15	0.96	0.01
Cr (223)	F_{TM}	98.68	2.02	0.98	0.03
	F_{CE}	91.11	5.84	0.91	0.06
	$F_{\mathrm{TM+CE}}$	99.57	1.30	0.99	0.02
	aSLATM	82.04	6.48	0.81	0.06
	$F_{\mathrm{TM+CE+aSLATM}}$	98.68	2.02	0.98	0.03
Mn (332)	F_{TM}	99.11	1.36	0.99	0.02
	F_{CE}	97.30	2.09	0.97	0.02
	$F_{\mathrm{TM+CE}}$	99.11	1.36	0.99	0.02
	aSLATM	95.51	3.30	0.95	0.04
	$F_{\mathrm{TM+CE+aSLATM}}$	98.81	1.96	0.99	0.02
Fe (563)	F_{TM}	67.33	2.34	0.56	0.04
	F_{CE}	86.68	3.99	0.86	0.04
	$F_{\mathrm{TM+CE}}$	96.09	3.26	0.96	0.03
	aSLATM	76.19	2.93	0.74	0.03
	$F_{\mathrm{TM+CE+aSLATM}}$	95.91	1.60	0.95	0.02
Co (418)	F_{TM}	82.29	4.94	0.77	0.05
	F_{CE}	86.12	4.38	0.85	0.04
	$F_{\mathrm{TM+CE}}$	97.13	1.78	0.97	0.01
	aSLATM	82.52	4.63	0.81	0.05
	$F_{\mathrm{TM+CE+aSLATM}}$	94.27	3.06	0.94	0.03
Ni (527)	F_{TM}	55.99	1.79	0.42	0.04
	F_{CE}	90.71	1.76	0.90	0.02
	$F_{\mathrm{TM+CE}}$	98.48	0.76	0.98	0.01
	aSLATM	89.96	2.90	0.89	0.03
	$F_{\mathrm{TM+CE+aSLATM}}$	97.73	2.03	0.98	0.02

Table S14: 43 complexes whose random forest model using $F_{\text{TM+CE}}$ results in incorrect prediction. OS: metal oxidation state, N_d: the number of d electrons, r_{rel}: relative metal radius, S_{DFT}: DFT computed ground state spin, S_{Exp}: experimentally characterized spin state, S_{ML}: predicted ground state spin by random forest model using $F_{\text{TM+CE}}$ trained on the TM-GSspin dataset, Prob.: ML prediction probability. If S_{Exp} was not found in the literature, it is indicated with a hyphen. S_{DFT} that does not match S_{Exp} is displayed in red, indicating that the ML model's reference data is incorrect. SCO indicates a spin-crossover complex.

	refcode	metal	OS	N_d	coordination geometry	coordinating atoms	r_{rel}	S_{DFT}	S_{Exp}	S_{ML}	Prob.
1	NAFNEO	Cr	2	4	Octahedral	Cl, Cl, P, P, P, P	0.958	3	–	5	0.53
2	NAQQAY	Mn	2	5	Octahedral	C, C, P, P, P, P	0.868	2	2	1	0.42
3	WUYFIH	Mn	3	4	Octahedral	C, C, C, C, C, C	0.911	3	–	5	0.70
4	NUNWUP	Fe	0	6	Tetrahedral	C, C, P, P	0.798	3	–	1	0.89
5	GAMZEA	Fe	2	6	Square planar	C, C, P, P	0.944	3	3	5	0.77
6	GAMZAW	Fe	2	6	Square planar	C, C, P, P	0.938	3	3	5	0.67
7	NENNEA	Fe	2	6	Square pyramidal	N, N, N, O, O	1.014	3	–	5	1.00
8	PEPPEI	Fe	2	6	Square pyramidal	P, P, P, Se, Se	0.902	3	5	1	0.52
9	DOQRAC	Fe	2	6	Octahedral	N, N, N, N, N, N	1.073	1	SCO	5	1.00
10	MAFECL	Fe	2	6	Octahedral	Cl, Cl, Cl, Cl, Cl, Cl	1.037	5	–	1	0.69
11	MAKBUW	Fe	3	5	Square planar	N, N, N, N	0.901	4	4	6	0.92
12	BOWYUJ	Fe	3	5	Tetrahedral	Br, Br, Br, Br	0.861	6	–	2	0.56
13	FIMPOH	Fe	3	5	Tetrahedral	N, P, P, P	0.849	2	2	6	0.98
14	RAKTAA	Fe	3	5	Square pyramidal	Cl, Cl, N, N, N	0.991	6	–	4	0.72
15	DADJEX	Fe	3	5	Square pyramidal	C, Cl, Cl, N, N	0.987	4	–	6	0.93
16	HOGHEQ	Fe	3	5	Square pyramidal	C, N, N, N, N	0.924	2	2	4	1.00
17	TIQZIE	Fe	3	5	Square pyramidal	N, N, N, N, O	0.982	4	4	6	1.00
18	QITWUO	Fe	3	5	Square pyramidal	N, N, N, N, O	0.99	4	–	6	0.78
19	QOCDUI01	Fe	3	5	Square pyramidal	Br, N, N, N, N	0.96	4	4	2	0.57
20	YITRUO	Fe	3	5	Square pyramidal	P, P, P, S, S	0.885	2	2	4	0.56
21	ALALAA	Fe	3	5	Square pyramidal	C, N, N, N, N	0.962	2	2	4	0.93
22	UCIVOT	Fe	3	5	Square pyramidal	O, O, O, O, O	0.97	6	6	4	0.65
23	EWOBEX	Fe	3	5	Square pyramidal	Br, C, N, N, N	0.972	4	4	6	0.98
24	OLUYEB	Fe	3	5	Octahedral	Cl, N, N, N, N, N	1.004	2	–	6	0.90
25	CIXXAJ	Co	1	8	Tetrahedral	P, P, P, Se	0.907	3	3	1	0.56
26	ROBXEM	Co	2	7	Tetrahedral	I, I, I, I	0.958	4	–	2	0.54
27	RAWRUF	Co	2	7	Square pyramidal	N, N, N, N, N	1.075	4	4	2	0.50
28	YUBZUR	Co	2	7	Square pyramidal	N, N, N, N, N	1.072	2	4	4	0.86
29	SEMROU	Co	2	7	Square pyramidal	Cl, Cl, N, N, N	1.07	4	–	2	0.64
30	RUMHAI	Co	2	7	Octahedral	N, N, P, P, S, S	1.092	2	4	4	0.57
31	KAXMIF	Co	3	6	Trigonal planar	N, N, N	0.921	5	5	3	0.48
32	ABATUT	Co	3	6	Trigonal planar	N, N, N	0.848	1	5	5	0.56
33	QOXZAG	Co	3	6	Seesaw	N, N, N, N	0.902	1	5	3	0.58
34	QOXZEK	Co	3	6	Tetrahedral	N, N, N, N	0.884	1	5	3	0.78
35	WATSUF	Co	3	6	Square pyramidal	C, N, N, N, N	0.952	1	1	3	0.56
36	LADCOI	Co	3	6	Trigonal bipyramidal	Cl, N, N, N, N	0.954	3	3	1	0.57
37	JARKIX	Ni	2	8	Linear	S, S	0.906	3	3	1	0.90
38	YOLDAG	Ni	2	8	T-shaped	C, N, N	0.944	1	1	3	0.58
39	YUJZAF	Ni	2	8	Square pyramidal	N, N, S, S, S	1.058	1	–	3	1.00
40	ENUSEN	Ni	2	8	Square pyramidal	C, C, N, N, N	1.052	1	1	3	0.99
41	PEMENI	Ni	2	8	Square pyramidal	N, N, O, P, P	1.046	1	1	3	1.00
42	AACANI11	Ni	2	8	Square pyramidal	N, N, O, O, O	1.046	3	–	1	0.70
43	URIRUL	Ni	2	8	Octahedral	I, I, S, S, S, S	1.148	1	–	3	1.00

References

(1) Vela, S.; Laplaza, R.; Cho, Y.; Corminboeuf, C. cell2mol: encoding chemistry to interpret crystallographic data. *npj Comput. Mater.* **2022**, *8*, 188.

(2) Butin, K. P.; Beloglazkina, E. K.; Zyk, N. V. Metal complexes with non-innocent ligands. *Russ. Chem. Rev.* **2005**, *74*, 531.

(3) Bernuz Fito, E. Cosymlib: a Python library for continuous symmetry measures and its application to problems in structural chemistry. **2021**,

(4) Cirera, J.; Ruiz, E.; Alvarez, S. Continuous shape measures as a stereochemical tool in organometallic chemistry. *Organometallics* **2005**, *24*, 1556–1562.

(5) Vogiatzis, K. D.; Polynski, M. V.; Kirkland, J. K.; Townsend, J.; Hashemi, A.; Liu, C.; Pidko, E. A. Computational approach to molecular catalysis by 3d transition metals: challenges and opportunities. *Chem. Rev.* **2018**, *119*, 2453–2523.

(6) Cordero, B.; Gómez, V.; Platero-Prats, A. E.; Revés, M.; Echeverría, J.; Cremades, E.; Barragán, F.; Alvarez, S. Covalent radii revisited. *Dalton Trans.* **2008**, 2832–2838.

(7) Huang, B.; von Lilienfeld, O. A. Quantum machine learning using atom-in-molecule-based fragments selected on the fly. *Nat. Chem.* **2020**, *12*, 945–951.

(8) Christensen, A. S.; Faber, F.; Huang, B.; Bratholm, L.; Tkatchenko, A.; Muller, K.; von Lilienfeld, O. QML: A Python toolkit for quantum machine learning. **2017**,
